# Triangular prism-shaped β-peptoid helices as unique biomimetic scaffolds

**DOI:** 10.1038/ncomms8013

**Published:** 2015-05-06

**Authors:** Jonas S. Laursen, Pernille Harris, Peter Fristrup, Christian A. Olsen

**Affiliations:** 1 Department of Chemistry, Technical University of Denmark, Kemitorvet 207, DK-2800 Kongens Lyngby, Denmark

## Abstract

β-Peptoids are peptidomimetics based on *N*-alkylated β-aminopropionic acid residues (or *N*-alkyl-β-alanines). This type of peptide mimic has previously been incorporated in biologically active ligands and has been hypothesized to be able to exhibit foldamer properties. Here we show, for the first time, that β-peptoids can be tuned to fold into stable helical structures. We provide high-resolution X-ray crystal structures of homomeric β-peptoid hexamers, which reveal right-handed helical conformations with exactly three residues per turn and a helical pitch of 9.6–9.8 Å between turns. The presence of folded conformations in solution is supported by circular dichroism spectroscopy showing length- and solvent dependency, and molecular dynamics simulations provide further support for a stabilized helical secondary structure in organic solvent. We thus outline a framework for future design of novel biomimetics that display functional groups with high accuracy in three dimensions, which has potential for development of new functional materials.

## Abstract (web summary)

β-Peptoids are based on *N*-alkylated β-aminopropionic acid residues, which have been inspired by the more intensely studied peptoids and β-peptides. Here, the authors report X-ray crystal structures of oligomeric β-peptoids demonstrating secondary structures with this backbone.

## Graphical abstract

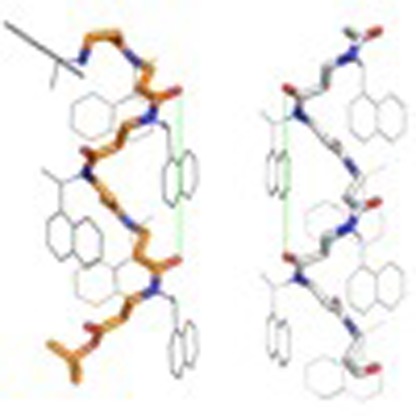

The ability to mimic or complement native protein and peptide folds using non-natural oligomers endowed with resistance towards proteolysis holds promise for the design of chemotypes with valuable applications in medical and materials sciences. Oligomeric architectures able to adopt well-defined folding patterns have been coined foldamers^1^ and several peptidomimetic designs have demonstrated this type of behaviour^2^,^3^,^4^,^5^, including β-peptides^6^,^7^,^8^ and peptoids (*N*-alkylglycines)^9^,^10^. The β-peptoids, first described as dimers and trimers by Hamper *et al.*^11^, combine the structural features of the two latter mentioned foldamers, and have been suggested by theoretical methods to be able to adopt helical conformations^12^. Bulky *N*-alkyl side chains with chirality in the α-position have been shown to induce helical secondary structures in α-peptoids with alternating handedness depending on stereochemical configuration^13^,^14^,^15^, and *cis-*amide-inducing α-chiral side chains have also been shown to induce helicity in aromatic oligoamides^16^. However, circular dichroism (CD)-spectroscopical evaluation of oligomers containing β-peptoid units with α-chiral side chains have proven inconclusive thus far^17^,^18^, and high-resolution structures have been lacking, except for head-to-tail cyclized species^19^. Nevertheless, β-peptoid units have been incorporated in oligomers showing non-haemolytic antimicrobial^20^,^21^,^22^,^23^,^24^, antiplasmodial^25^ and cell-penetrating^26^ activities, as well as excellent stability towards proteolytic enzymes^22^.

Inspired by studies performed using α-peptoid model systems^27^,^28^, the tunability of the *cis–trans*-amide bond equilibria of the tertiary amide bonds in β-peptoids have also been investigated^29^,^30^, and building on these insights, here we design β-peptoid oligomers containing *N*-(*S*)-1-(1-naphthyl)ethyl side chains for structural evaluation, and report the first high-resolution X-ray crystal structures of helical β-peptoid oligomers, which should enable informed, structure-based design of foldameric materials of this type in the future.

## Results

### Compound design

**Figure 1:**
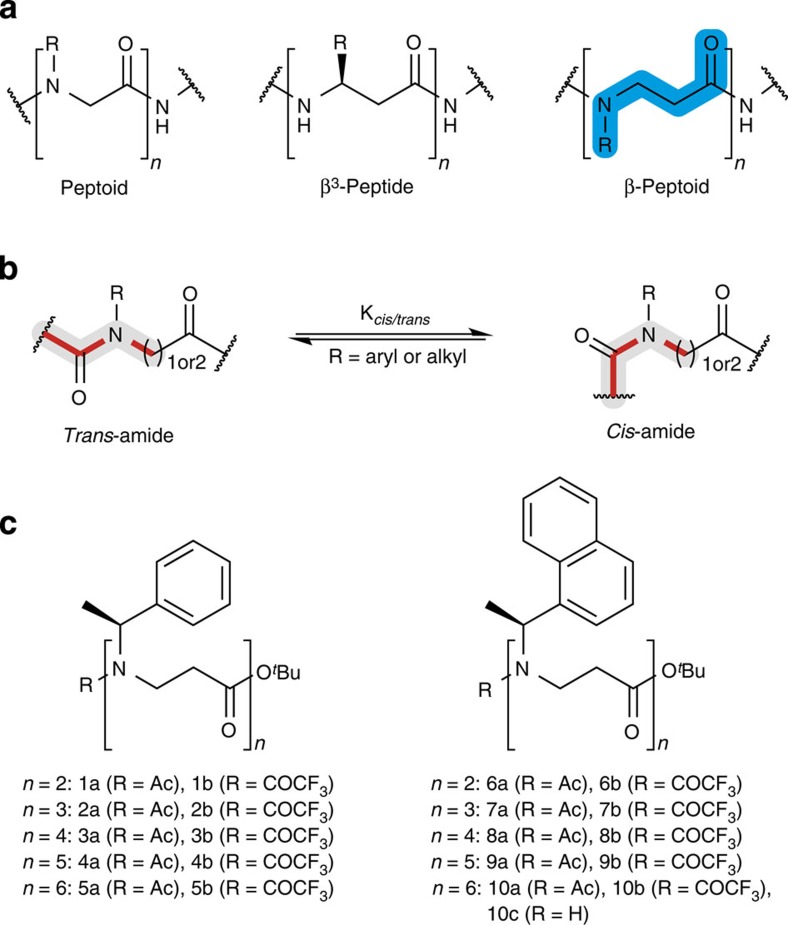
Chemical structures. (**a**) Generic structures of the backbone architectures of peptoids (that is, α-peptoids), β^3^-peptides (β^2^- and disubstituted β-peptides are not shown) and β-peptoids. (**b**) Depiction of the equilibrium of *transoid* and *cisoid* amide conformations in peptoid residues. (**c**) β-Peptoid oligomers prepared for structural and spectroscopic evaluation in this study.

Since high-resolution structures of α-peptoids have been successfully obtained for oligomers of just 4–5 residues in length, we envisioned that a hexamer might be sufficient to acquire stabilized folding. Two different α-chiral side chains were chosen (Fig. 1c): the *N*-(*S*)-1-phenylethyl (**1**–**5**), which has been studied in both α- and β-peptoids, and the *N*-(*S*)-1-(1-naphthyl)ethyl (**6**–**10**), which was applied in an α-peptoid tetramer structure solved by X-ray crystallography^31^. The *N*-(*S*)-1-phenylethyl side chain, although shown to successfully induce helical conformations in α-peptoids^13^,^14^,^15^,^32^, does not induce significant control of the amide bond *cis–trans* equilibrium. It is therefore not entirely surprising that β-peptoid oligomers of this type have not previously been unambiguously shown to establish robust secondary structures. In recent monomer-based model systems, however, we found that the *trans*-amide conformation was preferred upon trifluoroacetylation of *N*-(*S*)-1-phenylethyl monomers^30^. The effect of this substitution was therefore included in the present investigation (**1b**–**5b**) along with the acetylated control compound series **1a**–**5a**. The second series contained the *N*-(*S*)-1-(1-naphthyl)ethyl side chain (**6a**–**10d**), which was first introduced in β-peptoids^33^, and later found to strongly induce *cis*-amide conformations in model systems^28^,^30^ as well as in helical^31^ and ribbon-shaped^34^ α-peptoids.

**Figure 2:**
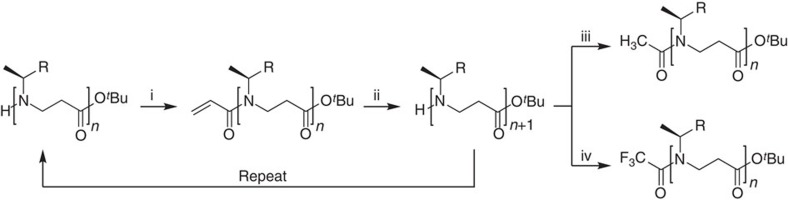
Oligomerization and N-terminal functionalization of β-peptoids. Reagents and conditions: (i) acryloyl chloride, NEt_3_, THF, 0 °C; (ii) (*S*)-1-phenylethylamine or *(S*)-1-(1-naphthyl)ethylamine, MeOH, 50 °C; (iii) AcCl, NEt_3_, 0 °C; (iv) (CF_3_CO)_2_O, NEt_3_, CH_2_Cl_2_, 0→20 °C.

All compounds were prepared by subunit synthesis in solution, essentially as previously described^35^ (Fig. 2). For the considerably sterically congested naphthyl-containing compounds, in particular, we found the necessary reaction times to increase with the growing length of the oligomer, and found that fragment-based coupling may be necessary for efficient preparation of higher oligomers^20^ (further synthetic considerations *vide infra*).

### *Cis–trans*-amide rotamers

^1^H nuclear magnetic resonance (NMR) spectra of the oligomers exhibited significant signal overlapping due to the identical side chains throughout each series as would be expected. We therefore focused on the shifts corresponding to the side chain methyne protons to determine overall *cis–trans*-amide bond ratios (Supplementary Figs 1–5; Table 1). NMR data showed no significant trend on the overall *cis–trans* ratios for the compounds in the *N*-(*S*)-1-phenylethyl series (**1**–**5**). In the *N*-(*S*)-1-(1-naphthyl)ethyl series (**6**–**10**), however, increases in *K*_*cis–trans*_ values could be observed upon elongation of the oligomers, as particularly evident for the constants determined for the hexamers in benzene (Table 1). Unfortunately, the two highest oligomers of this series were not sufficiently soluble in acetonitrile to obtain NMR spectra of sufficient quality, but especially for the trifluoroacetylated series, there appears to be a slight increase in *K*_*cis–trans*_ from dimer to tetramer. Taken together, these values may indicate propensity for the oligomers to adopt length- as well as solvent-dependent secondary structures, although high-resolution measurements are required to determine whether this is the case.

### X-ray crystallography

Gratifyingly, we were able to crystallize compounds **10a**, **10b** and **10c** by slow evaporation of MeOH–CHCl_3_ or benzene solutions of the compounds. Diffraction-quality crystals were obtained for **10a** and **10c** from MeOH–CHCl_3_, and their structures were solved by X-ray single-crystal structure determination to 1.05 and 1.00 Å resolution, respectively (see Supplementary Figs 6–8 for additional representations).

**Figure 3:**
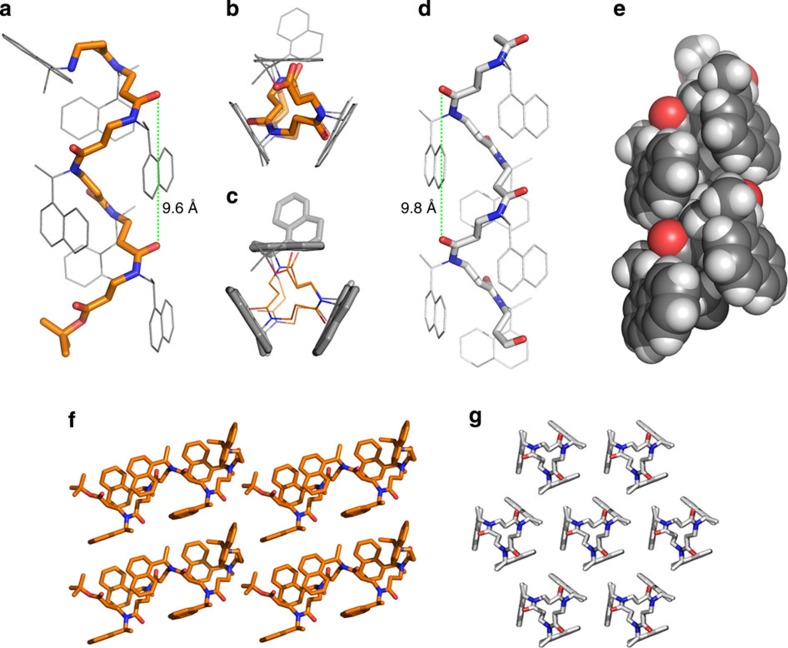
X-ray crystal structures. (**a**) Side view of compound **10c**. (**b**,**c**) End views of compound **10c** from the C terminus. The backbone is shown in orange and hydrogen atoms are omitted for clarity. (**d**) Compound **10a** showing the backbone helix as sticks and side chains as lines. (**e**) Spacefilling representation of compound **10a** showing the packing of the naphthyl groups along the phases of the triangular prism-shaped conformation. (**f**) Crystal packing of compound **10c** viewed perpendicular to the helical axis. (**g**) Crystal packing of compound **10a** viewed down the helical axis to demonstrate the highly ordered arrangement. Hydrogen atoms are omitted for clarity except for the spacefilling representation in **e**. Representations of X-ray crystal structures were prepared using the PyMOL Molecular Graphics System, Schrödinger, LLC.

The N-terminally non-acylated compound **10c** was solved first and revealed a helical conformation of precisely three residues per turn and a pitch of ∼9.6 Ångström, with only the N-terminal naphthyl side chain twisted away from the helical axis (Fig. 3a; Supplementary Data 1). The remaining naphthyl groups were highly organized along each of the three faces of the helix to give an equilateral triangle when viewed down the helical axis (Fig. 3b,c). The main helix thus adopts a triangular prism shape, not taking the N-terminal side chain into consideration. We envisioned, based on our monomer studies, that acetylation of the N terminus would also force this amide into a *cisoid* configuration, and hoped that this would direct the final side chain into alignment along the third face of the molecule. The X-ray crystal structure of compound **10a** indeed confirmed this (Fig. 3d; Supplementary Data 2), as this compound crystallized in the *R*3 space group with only one -CH_2_CON(1-(1-naphthyl)ethyl)CH_2_- fragment in the asymmetric unit. Thus, the crystal symmetry generates infinitely long, parallel chains, and this renders the *t-*butyl group invisible, due to the 1:6 ratio of *t*-butyl to 1-(1-naphthyl)ethyl.

This results in crystal packing, where the helical segments are arranged in a head-to-tail manner to give regularly elongated triangular threads (Fig. 3g) with a helical pitch of 9.8 Å (Fig. 3d). To reveal the complete molecule, including the N-terminal-acetamido and C-terminal *t*-butyl ester groups, data sets were collected on several crystals, but attempts to solve the structure in *P*1 space group were unsuccessful. This packing issue was not observed for compound **10c** due to the positioning of the N-terminal side chain, and therefore enabled visualization of the full structure including the C-terminal *t*-butyl (Fig. 3f).

The torsion angles in the two helices are very similar (Table 2), and the extended structures compare reasonably well with one of the helical conformations previously suggested by density functional theory calculations, albeit with a ∼20-degree difference in the *ω* and *θ* angles (Supplementary Table 1)^12^. To the best of our knowledge, however, the present work provides the first experimental demonstration of the existence of this helical type. Most previously determined helical conformations of a β-peptidic nature have been shown to contain stabilizing hydrogen bond networks^7^, and investigation of oligomers of homologated proline residues indicated *trans*-amide-containing conformations^36^. Our structures are thus unique to the field of foldamers. The highly ordered, tight packing of the side chains along the three faces of the helical axis (Fig. 3e) combined with their strong *cis*-amide-inducing properties^30^, suggests that this type of side chain provides a particularly strong stabilization of this novel secondary-structure motif.

Furthermore, intramolecular distances indicate CH–π interactions between the backbone methylene group and side chain naphthyl groups, which may also contribute to stabilization and protection of the helical backbone from solvation (Supplementary Fig. 9; Supplementary Table 2). This is in stark contrast to the X-ray crystal structure reported for an α-peptoid tetramer containing the same side chains, where the naphthyl groups pointed away from the helical axis^31^. In agreement with that structure, however, as well as the findings from our previous monomer investigations, we did not see evidence for stabilizing n→π*_Ar_ interactions in our crystal structure. On the other hand, our *cis-*amide conformations were in agreement with the recently described n→σ* orbital overlap between carbonyl and side chain methyne atoms observed for peptoids^37^.

### CD and fluorescence spectroscopy

**Figure 4:**
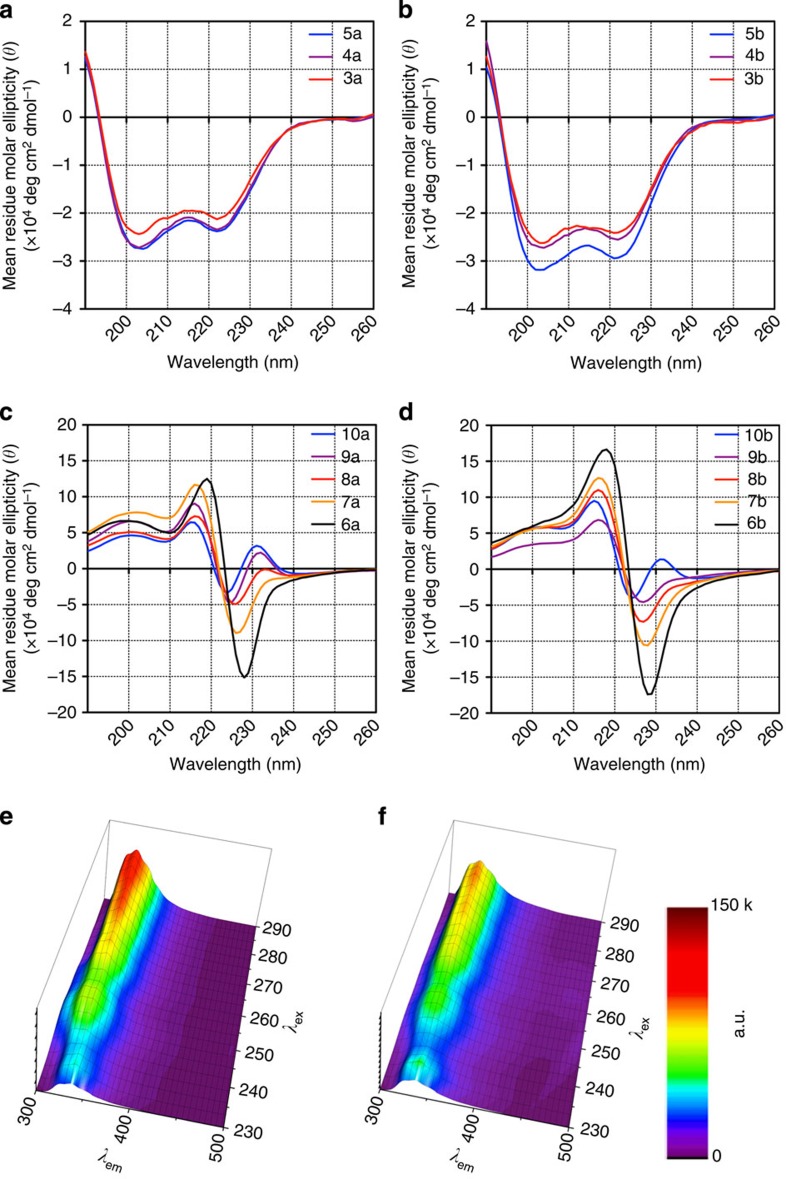
CD and fluorescence spectra. (**a**) CD spectra of compounds **3a**–**5a**; (**b**) CD spectra of compounds **3b**–**5b**; (**c**) CD spectra of compounds **6a**–**10a**; and (**d**) CD spectra of compounds **6b**–**10b** measured at room temperature in acetonitrile (60 μM). (**e**) Fluorescence emission spectra recorded for compound **10a** in acetonitrile or (**f**) in MeOH–acetonitrile (3:2). Spectra were obtained at room temperature using 60-μM solutions, and the intensities are shown in arbitrary units (a.u.).

We then evaluated the compounds by CD spectroscopy. The spectra obtained for the acetylated *N*-(*S*)-1-phenylethyl series (Fig. 4a,b) were similar to those previously reported for β-peptoids containing this side chain, whereas the *N*-(*S*)-1-(1-naphthyl)ethyl series (**6a**–**10a**) revealed a minimum at 224–228 nm and a maximum at 215–218 nm (Fig. 4c). At first glance, we were surprised that these Cotton effects decreased with increasing length of the oligomer. However, both the minimum and the maximum were present in the CD spectrum of an acetyled monomer and not of a non-acetylated monomer (Supplementary Fig. 10), which indicates that these signals are not related to secondary-structure formation but rather a signature of the particular naphthyl-containing amide motif itself. Starting at the tetramer length (**8a**), on the other hand, a positive signal appeared near 232 nm. This signal intensified with growing length of the oligomers, suggesting that this peak may be indicative of length-dependent secondary-structure formation.

We also obtained CD spectra of the trifluoroacetylated oligomers, which exhibited the same overall spectral shapes (Fig. 4d). Notably, six residues were necessary to obtain a positive signal at 232 nm in this series, suggesting that trifluoroacetylation has a negative effect on secondary-structure formation in acetonitrile. In our monomer study, we observed stabilization of the *cis*-amide conformation upon trifluoroacetylation compared with acetylation^30^, but this apparently does not translate into a stabilizing effect on the oligomer secondary structure. This may be explained by a steric clash between the bulkier trifluoroacetyl group and the first backbone methylene group at the N terminus.

To assess whether the positive signal at 232 nm is indicative of folding, we also collected CD spectra of both **10a** and **10b** at temperatures in the range 20–75 °C (Supplementary Fig. 11). The gradual decrease in signal intensity at 232 nm upon heating was indeed in accordance with temperature-mediated denaturation. Importantly, the remaining spectral shape was not affected significantly, which is in accordance with our hypothesis that this is not related to secondary structure. Furthermore, spectra recorded upon cooling were identical to those obtained before heating, indicating refolding of the structures (Supplementary Fig. 12). In addition, we investigated the effect of solvent polarity by obtaining CD spectra of compounds **9a**, **10a** and **10b** in the presence of varying amounts of MeOH (Supplementary Fig. 13). These spectra also showed a decrease in the signal at 232 nm, which is in agreement with denaturation of our structure at gradually increasing MeOH concentrations. Finally, we obtained CD spectra at varying concentrations of **10a** (Supplementary Fig. 14), which showed a slight increase of the signal at 232 nm upon dilution. While this was somewhat surprising to us, it suggests that aggregation does not play a role in the formation of secondary structure.

Aggregation of naphthyl-containing side chains in peptoids was recently shown to give rise to CD signals that correlated with fluorescent excimer bands^38^; thus, to rule out a similar origin of the maxima at 232 nm, we subjected oligomer **10a** and dimer **6a** to fluorescence measurements. In acetonitrile, the hexameric oligomer displayed a single maximum at ∼336 nm (Fig. 4e) and no change in spectral shape was observed upon addition of methanol, which might promote aggregation due to poorer solubility (Fig. 4f). An excimer band would be expected to appear at a longer wavelength in the emission dimension if aggregates formed, as previously reported for peptoids containing the same type of side chains (392 nm)^38^. This was not the case for either compound **10a** or the dimer **6a** (Supplementary Fig. 15), suggesting that the CD maxima observed at 232 nm for our longer oligomers are not a result of excimer effects caused by naphthyl group aggregation.

### Structure–folding relationships

**Figure 5:**
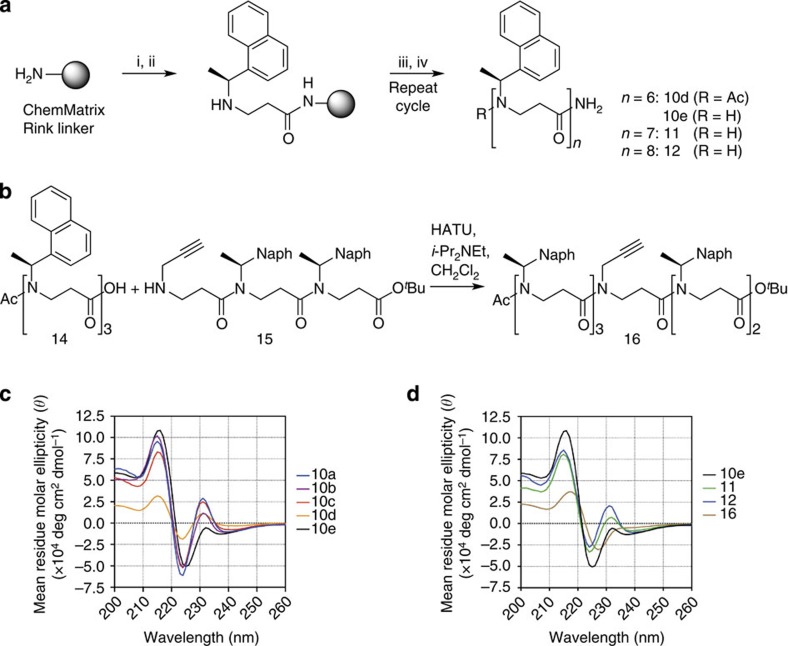
Synthesis and CD spectra of additional analogues. (**a**) Solid-phase synthesis of homo-oligomers. Reagents and conditions: (i) acryloyl chloride (6 equiv), *i*-Pr_2_NEt (12 equiv), DMF, 2 h, RT; (ii) (*S*)-1-(1-naphthyl)ethylamine (6 equiv), MeOH, 16 h, 50 °C; (iii) only for compound **10d**: AcCl (6 equiv), *i*-Pr2NEt (12 equiv), DMF, 2 h, RT; (iv) trifluoroacetic acid CH_2_Cl_2_ (1:1). (**b**) Fragment coupling in solution to give hetero-oligomeric hexamer **16** (49% isolated yield). Reagents and conditions: HATU (1.1 equiv), *i*-Pr_2_NEt (2 equiv), CH_2_Cl_2_, 16 h, RT. (**c**,**d**) CD spectra measured in MeCN (60 μM).

Acylation of the N-terminus appeared to have a pronounced effect on folding propensity as indicated by the two X-ray crystal structures (**10a** versus **10c**), as well as the CD spectra (for example, **9a** versus **9b**). These observations in addition to an interest in being able to introduce additional functionalities in future investigations of the applicability of these oligomers as scaffolds for multivalent display prompted us to prepare modified analogues of **10a**. Owing to the challenges associated with synthesis of oligomers by subunit assembly in solution above the hexamer length, we explored two additional strategies to prepare analogues. First, we attempted to transfer the subunit approach to solid support using a ChemMatrix resin with a Rink linker to give oligomers containing C-terminal amides (Fig. 5a). Due to increasing solubility difficulties encountered during purification for acetylated oligomers upon growing length (that is, **10d**), we prepared oligomers with free N termini by solid-phase synthesis (**10e**, **11** and **12**). This strategy afforded sufficient material for CD spectroscopic evaluation, but the crude purities observed by analytical high-performance liquid chromatography (HPLC) were not appropriate for synthesis of hetero-oligomers (Supplementary Fig. 16). Thus, it was decided to explore a fragment-based coupling strategy to introduce an alkyne-containing side chain, which could be easily functionalized by Cu(I)-catalyzed Huisgen azide-alkyne cycloaddition^39^,^40^ after oligomerization. Indeed, compound **16** was obtained in good yield by solution-phase coupling of two trimer building blocks (Fig. 5b), showing that β-peptoid oligomers may be obtained efficiently by fragment coupling in solution as was also previously demonstrated on solid phase^20^.

This additional collection of compounds was evaluated by CD spectroscopy to investigate structural influence on the hypothesized structure indicating Cotton effect at ∼232 nm (Fig. 5c). As evident from the series of different hexamers **10a**–**e**, the combination of N-terminal acetylation and a C-terminal *tert*-butyl ester of compound **10a** gave rise to the most robust signal at 232 nm. Compound **10c**, containing a *tert*-butyl group at the C-terminus and a free N-terminus, displayed an intense Cotton effect at 232 nm similar to compound **10a** and more intense than compound **10d**, which suggests that the bulky C-terminal esterification is more important for helix formation and/or stabilization than N-terminal acetylation (Fig. 5c).

Compound **10e**, which has a C-terminal amide and free N-terminus, exhibited the lowest intensity at 232 nm in this series. However, this signal was gradually rescued upon elongation to heptamer (**11**) and octamer (**12**) length, indicating cooperative length-dependent secondary-structure formation (Fig. 5d).

Finally, we were interested in investigating whether side chains could be mutated to other residues than *N*-(*S*)-1-(1-naphthyl)ethyl with retention of structural integrity, which would allow for further functionalization of these novel scaffolds. We therefore prepared compound **16** as described above. The CD spectrum of this compound, however, indicated a complete collapse of the secondary structure (Fig. 5d) by exhibiting a shape similar to trimer **7a**. While this supports our previously mentioned hypothesis that tight packing of the naphthyl side chains along the helix serves to stabilize the structure, it also shows that more elaborate synthetic efforts are required to achieve funtionalized oligomers with retention of the helical structure. We therefore envision that functional groups should be introduced at the methyl position of the side chains instead.

CD spectroscopy of peptide analogues, however, is not sufficient to derive specific secondary structures^41^, and although we find it highly likely that our oligomers adopt helical conformations in solution based on our collective results including X-ray, we stress that our current NMR and CD data do not unambiguously verify the presence of such secondary structures. Therefore, we also performed molecular dynamics simulations to address the folding propensity of hexamer **10a** in solution.

### Molecular dynamics simulations

To lend further theoretical support to the presence and stability of helical structures in solution, we carried out a series of molecular dynamics simulations on hexamer **10a** using a graphics processing unit (GPU)-accelerated version of Desmond (v 3.9). The hexamer was built in a helical conformation in maestro v. 9.8 (Schrödinger, LLC, New York, NY, 2014) and treated as previously described^42^ (see the Methods section for details).

To investigate the influence of temperature, we carried out a series of 250-ns simulations at 200, 250, 300, 350 and 400 K, respectively (Supplementary Figs 17–21). These simulations revealed significant temperature dependence with the ‘low-temperature' regimes (200 and 250 K) showing only few conformations, while the structural ensemble displayed high heterogeneity in the high-temperature regimes (350 and 400 K). In the simulation carried out at 200 K, the hexamer was dominated by a meta-stable folded conformation (root mean squared deviation (r.m.s.d.) ∼4 Å), which was populated for more than 100 ns (Supplementary Fig. 17). When the temperature was raised to 250 K, the structure alternated between the folded and unfolded conformation significantly faster with the meta-stable conformations having lifetimes of 10–30 ns (Supplementary Fig. 18).

**Figure 6:**
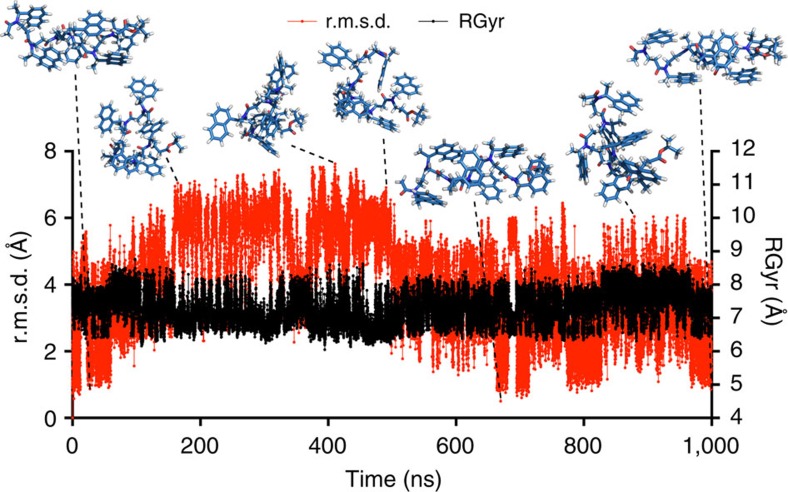
Molecular dynamics simulation of compound **10a** in acetonitrile. The axes show root mean square deviation (r.m.s.d.) and gyrational radius (RGyr) during a 1-μs molecular dynamics (MD) simulation at 300 K. Representative conformations are shown to illustrate the folded and unfolded states of the helical structure throughout the simulation. The simulation is uploaded as Supplementary Movie 1.

**Figure 7:**
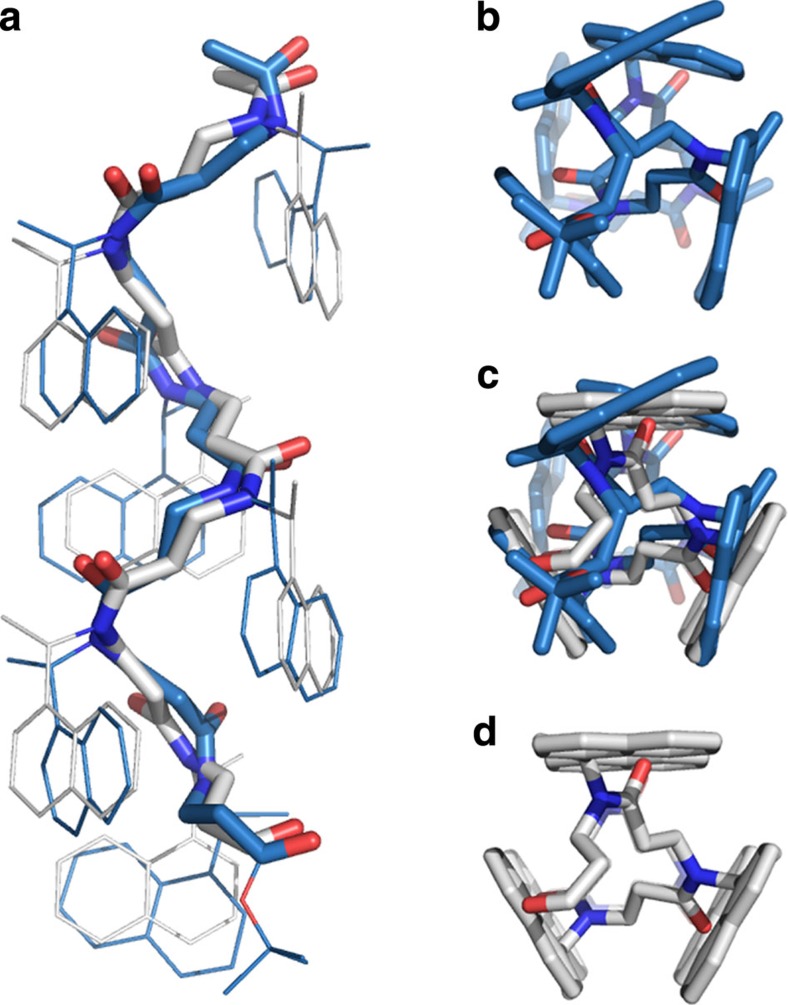
Overlays of conformations of compound 10a. (**a**) Side view of the X-ray crystal structure shown in light grey and the conformation after 1 μs MD simulation shown in blue. Only the backbones are shown as sticks to highlight the similarity. (**b**–**d**) End views shown as stick representations. The r.m.s.d. was calculated for all backbone atoms in structural overlays prepared using the ‘pair-fit' command in MacPyMOL, Schrödinger Inc.

The simulation at 300 K exhibited intermediary fluctuations in r.m.s.d. and gyrational radius (RGyr), with conformations similar to the starting point (r.m.s.d.∼2 Å) appearing throughout the simulation (Supplementary Fig. 19). To investigate conformational space at this temperature in more detail, we also performed a longer (1 μs) simulation where low r.m.s.d. and high Rgyr, which is in agreement with helical conformation, were observed within the first 60-ns interval (Fig. 6). Then bent conformations with r.m.s.d.s of up to >6 Å and slightly decreased RGyr (<7 versus 7.5 Å for the helical structure) were observed. A pattern of unfolding and refolding occurred throughout the simulation, with the conformations gradually diverging from the initial X-ray structure until approximately the 500-ns time point. Interestingly, significantly lower r.m.s.d. values reappeared in the later part of the simulation, and overlaying one of the final structures with the X-ray crystal structure of compound **10a** revealed an r.m.s.d. for all backbone atoms of just 0.837 Å (Fig. 7).

## Discussion

We report the first examples of high-resolution structures of linear oligomeric β-peptoids, which are also the longest structures of any peptoid oligomer determined by X-ray crystallography to date. These novel helical structures show definitively that the β-peptoids qualify as a valid addition to the already rich ensemble of foldamer designs. Our crystal structures revealed highly regular equilateral triangular prism-shaped conformations in the solid state, which was achieved by synthesis of homomeric hexamers containing the highly *cis*-amide-inducing side chain, (*S*)-1-(1-naphthyl)ethylamine. Furthermore, ^1^H NMR and CD spectroscopic data supported the existence of length-, solvent- and temperature-dependent secondary structures in solution in organic solvents. Although the determination of high-resolution structures of oligomeric β-peptoids in solution will be an interesting future challenge, we here performed molecular dynamics simulations in acetonitrile, which provided further evidence for the formation of helical conformations in solution. Taken together, we demonstrate control of β-peptoid folding for the first time, which now opens the possibility of taking advantage of β-peptoids in design of novel structurally well-defined biomimetic materials. Furthermore, a preliminary structure–folding relationship investigation suggested that a bulky ester substituent at the C-terminus is important for stable folding and, importantly, showed that mutation of an (*S*)-1-(1-naphthyl)ethylamine side chain to a readily functionalizable acetylene-containing side chain was incompatible with helicity, as indicated by CD spectroscopy. We are therefore currently investigating various strategies to introduce alternative functionalities through substitution at the methyl substituent position of our side chains to ensure structural integrity of these novel scaffolds. We envision that functionalized helical β-peptoids have potential applicability in biomedical research. Well-defined positioning of amino acid side chain functionalities in three dimensions is important for disruption of protein–protein interactions, which account for a number of notoriously difficult-to-inhibit drug targets. The structures provided herein may serve as useful abiotic scaffolds with proteolytic stability in such endeavours. Furthermore, we envision that helical β-peptoids may serve as scaffolds for highly accurate multivalent display of drug molecules in three dimensions to achieve increased binding affinities against targets with multiple binding sites.

## Methods

### General

All chemicals and solvents were analytical grade and used without further purification. Vacuum liquid chromatography (VLC) was performed on silica gel 60 (particle size 0.015−0.040 mm). Ultra high-performance liquid chromatography (UPLC)-mass spectrometry (MS) analyses were performed on a Phenomenex Kinetex column (1.7 μm, 50 × 2.10 mm) using a Waters Acquity ultra-HPLC system. A gradient with eluent I (0.1% HCOOH in water) and eluent II (0.1% HCOOH in acetonitrile) rising linearly from 0 to 95% of II during *t*=0.00–5.20 min was applied at a flow rate of 1 ml min^−1^. Preparative HPLC purification was performed on a C18 Phenomenex Luna column (5 μm, 100 Å, 250 × 20 mm) using an Agilent 1260 LC system equipped with a diode array ultraviolet detector and an evaporative light-scattering detector (ELSD). A gradient with eluent III (water−MeCN−TFA, 95:5:0.1) and eluent IV (0.1% TFA in acetonitrile) rising linearly from 45 to 95% of IV during *t*=5−35 min, and isocratically at 95% from *t*=35–55 min was applied at a flow rate of 20 ml min^−1^. Analytical HPLC was performed on a C18 phenomenex Luna column (3 μm, 100 Å, 150 × 4.60 mm) using an Agilent 1100 series system equipped with a diode array ultraviolet detector. A gradient using eluent III and eluent IV rising linearly from 0 to 80% of IV during *t*=2−10 min, then from 80 to 95% during *t*=10–27 min and finally isocratically at 95% IV from *t*=27–33 min was applied at a flow rate of 1 ml min^−1^. High-resolution LC-DAD-MS was performed on an Agilent 1,100 system equipped with a photodiode array detector (DAD) and coupled to a LCT orthogonal time-of-flight mass spectrometer (Waters-Micromass, Manchester, UK) with Z-spray electrospray ionization. Two-dimensional NMR spectra were recorded on a Bruker Ascend 400 at 400 MHz for ^1^H and 100 MHz for ^13^C. All spectra were recorded at 298 K. Heteronuclear single-quantum coherence spectra were recorded with a relaxation delay of 1.5 s before each scan, a spectral width of 4,800 × 16,600, collecting 4 FIDs and 1,000 × 256 data points. All spectra were recorded at 298 K. Chemical shifts are reported in p.p.m. relative to deuterated solvent peaks as internal standards (δH, C_6_D_6_ 7.16 p.p.m.; δC, C_6_D_6_ 128.06 p.p.m., δH, CD_3_CN 1.94 p.p.m.; δC, CD_3_CN 1.32 p.p.m.).

### General procedure for oligomerization of β-peptoids

The β-peptoids were oligomerized using a procedure closely resembling that reported by Taillefumier *et al.*^35^. Briefly, the β-peptoid (1.0 equiv) and NEt_3_ (1.2 equiv) were dissolved in THF (0.05 M) and cooled to 0 °C. Then acryloyl chloride (1.4 equiv) was added and the reaction was stirred for 1 h. The reaction mixture was filtered and the solids were washed with EtOAc. The combined filtrates were concentrated *in vacuo* yielding crude acrylamide. The crude acrylamide was dissolved in MeOH (0.1 M), the desired primary amine (2.0 eqiuv) was added and the reaction was stirred for 18 h at 50 °C. In case of the penta- and hexamer of compounds containing *N*-(*S*)-1-(1-naphthyl)ethyl side chains, additional amine (2.0 equiv) and stirring for 72 h were needed for the reaction to go to completion. Furthermore, the acrylamide of the *N*-(*S*)-1-(1-naphthyl)ethyl-containing pentamer was poorly soluble in MeOH, therefore MeOH–CH_2_Cl_2_ (1:3, 0.015 M) was used for the final aza-Michael step. Purification was performed using vacuum liquid silica gel chromatography applying a gradient of MeOH in CH_2_Cl_2_ from 0→5%.

### General procedure for N-terminal functionalization of β-peptoid oligomers

Acetylation of β-peptoid oligomers was achieved by dissolving the β-peptoid oligomer (1.0 eqiuv) in CH_2_Cl_2_ (0.02 M) at 0 °C and then adding Et_3_N (1.4 equiv) and acetyl chloride (1.2 equiv) under stirring for 1 h. Trifluoroacetylation of β-peptoid oligomers required addition of Et_3_N (4.0 equiv) and trifluoroacetic anhydride (5.0 equiv) to the β-peptoid solution, which was stirred for 1 h at 0 °C, followed by ambient temperature for 18 h. For both acetylated and trifluoroacetylated compounds, purification could be achieved by evaporation of the solvent *in vacuo* followed by preparative reversed-phase HPLC. Vacuum liquid silica gel chromatographic (VLC) purification of compounds **6a**–**10b** applying a gradient of 0.2% MeOH in CH_2_Cl_2_ from 0→5% was unsuccessful. However, crude **10a** and **10b** could be redisolved in *N,N*-dimethylformamide (DMF), and precipitated upon addition of water. The solid product contained only minor impurities, which could be removed using VLC with the above-mentioned gradient. All compounds were purified to >95% homogeneity, as judged by UPLC-MS and analytical HPLC (230 nm). HPLC traces for all oligomers are shown in Supplementary Figs 22 and 23. ^1^H NMR spectra are included as Supplementary Figs 24–44 and high-resolution mass spectrometry (HRMS) data are reported in Supplementary Table 3.

### Subunit synthesis of oligomers 10d, 10e, 11 and 12

A solution of acryloyl chloride (6 equiv) and *i*-Pr_2_NEt (12 equiv) in DMF (5 ml) were added to a Rink-amide-functionalized Chem-Matrix resin (1 equiv). After shaking for 3 h, the resin was drained and subsequently washed with DMF (3 × 3 ml), MeOH (3 × 3 ml), and CH_2_Cl_2_ (3 × 3 ml). Then a solution of (*S*)-1-(1-naphthyl)ethylamine (6 equiv) in MeOH (5 ml) was added to the resin that was shaken at 50 °C for 18 h, and then washed as described above. After nine coupling cycles, the crude product was cleaved from the resin using 50% TFA in CH_2_Cl_2_ for 2 × 30 min. Due to incomplete couplings it was possible to collect oligomers of 6–8 residues using preparative HPLC (Supplementary Fig. 16). All compounds were purified to >95% homogeneity as judged by UPLC-MS and analytical HPLC (230 nm). ^1^H NMR spectra are included as Supplementary Figs 45–48 and HRMS data are reported in Supplementary Table 4.

### Compound 14

The methylester-protected β-peptoid oligomer could be synthesized in the same manner as the *tert*-butylesters, starting from methylacrylate. The trimeric oligomer (514 mg, 0.68 mmol, 1 equiv) was dissolved in MeOH (7 ml), and 1 M aq. LiOH (2.7 ml, 2.7 mmol, 4 equiv) was added; the resulting mixture was stirred for 18 h at room temperature. The reaction mixture was poured into CH_2_Cl_2_ (100 ml) and washed with 1 M aq. HCl (100 ml), and the aqueous phase was then back extracted with CH_2_Cl_2_ (3 × 100 ml). The combined organic phases were dried (MgSO_4_), filtered and concentrated *in vacuo*. The compound was used without further purification. Yield: 450 mg (95%); UPLC-MS, *t*_R_=2.48 min, *m/z* [M+H]+ calcd for C_47_H_50_N_3_O_5_+, 736.4; found 736.9.

### Compound 15

The propargyl-containing β-peptoid oligomer could be synthesized by the general procedure described above, using propargyl amine in the third Michael addition. Purification was performed using vacuum liquid silica gel chromatography (2 × 6 cm, CH_2_Cl_2_–MeOH, 0.2% gradient from 0→5%) to give the product as a white solid. Yield: 819 mg (19% overall), UPLC-MS: *t*_R_=2.00 min, *m/z* 634.4 ([M+H]^+^, C_20_H_34_N_3_O_4_^+^, calcd 634.8).

### Compound 16

Compound **14** (219 mg, 0.32 mmol, 1 equiv), HATU (134 mg, 0.35 mmol, 1.1 equiv) and *i*-Pr_2_NEt (111 μl, 0.64 mmol, 2 equiv) were dissolved in CH_2_Cl_2_ (25 ml) and preincubated for 10 min at room temperature. Then, compound **15** (200 mg, 0.32 mmol, 1 equiv) was added and the resulting reaction mixture was allowed to stir for 2 h. The solvent was evaporated *in vacuo* and the crude compound was purified to >95% homogeneity using preparative HPLC. Yield 220 mg (49%). UPLC-MS: *t*_R_=3.37 min, *m/z* [M+H]^+^ calcd for C_83_H_88_N_7_O_7_^+^, 1,351.7; found 1,351.6. HPLC: *t*_R_=19.25 min (>95%). HRMS: *m*/*z* [M+Na]^+^ calcd for C_87_H_93_N_6_O_8_Na^+^, 1,373.7025; found, 1,373.7019 (ΔM=0.2 p.p.m.). See Supplementary Fig. 49 for a copy of the ^1^H NMR spectrum.

### Crystallization

Selected peptoid hexamers (∼30 mg) were dissolved in a minimum amount of CHCl_3_, and MeOH was added until precipitation could be observed; sufficient CHCl_3_ was then added to give clear solutions. The solutions were left for slow evaporation at room temperature, and crystrals were generally formed within a week. The crystals could be removed from the solvent and washed using MeOH. The hexamers (30 mg) also crystalized upon slow evaporation from benzene; however, these specimens were of insufficient quality for X-ray diffraction.

### X-ray crystallography

Several crystals of each compound were tested before suitable crystals were found. Measurements were performed at room temperature as cryo cooling turned out to destroy the crystals. Data collection was performed on an Agilent Supernova Diffractometer using CuKα radiation. For crystal **10a**, data to 1.00 Å resolution were included and for crystal **10c**, data to 1.05 Å resolution were included. Data were processed and scaled using the *CrysAlisPro* software (Agilent Technologies). Details of the data collection may be found in Supplementary Data 1 and 2.

Compound **10c** crystallized in space group *P*1 with one molecule in the asymmetric unit. The structure was solved using SHELXS and refined using SHELXL^43^. Due to the rather low resolution, it turned out, however, that geometric restraints had to be included in the refinement. All naphthyl groups were imposed with FLAT and DELU restraints. The N-terminal naphthyl group had rather large thermal parameters and was restrained further using RIGU and ISOR restraints. Furthermore, the *tert-*butyl group was restrained using DELU, and all six residues were restrained to be similar using a SAME command. In the rather large voids between the helices we observed disordered electron density. The three largest peaks were modelled as oxygen with occupancy of 0.5. Compound **10a**, on the other hand, crystallized in space group *R*3 with one peptoid residue in the asymmetric unit. The structure was solved using SHELXS and refined in SHELXL^43^. Also here, geometric restraints had to be included in the refinement and the naphthyl group was imposed with FLAT and DELU restraints. The peptoid oligomer was revealed when crystallographic symmetry was imposed. However, the crystal structure generates infinitely long chains and the *tert-*butyl group was not visible, although for every sixth naphthyl group a *tert-*butyl group must be present. Hydrogen atoms were included on ideal positions using riding coordinates for both crystals. The data quality did not allow for determination of the absolute configurations, which were imposed in accordance with the stereochemistry of the building blocks used in the synthesis.

### CD spectroscopy

Spectra were acquired with an Olis DSM 10 CD spectrophotometer (Olis Inc., Bogart, GA, USA), equipped with a Quantum Systems temperature control module, or on a Jasco 810 spectrometer equipped with a Julabo AWC 100 cooling unit. Spectra were obtained using 1-mm quartz cuvettes, and compound solutions in MeCN were prepared using dry weight of the lyophilized material followed by dilution to give the desired concentrations and solvent combination. The data are averages of 3–5 successive accumulations. Spectra were recorded in millidegree units, corrected for solvent contributions and normalized to mean residue ellipticity [*θ*]=100*ψ*/*lcn*, where *ψ* is the signal in millidegrees, *l* is the path length (0.1 cm), *c* is the concentration in mM and *n* is the number of peptoid amide bonds.

### Fluorescence scanning

Samples were prepared based on dry weight of lyophilized compounds **6a** and **10a**, respectively. Fluorescence was measured at ambient temperature in a black non-binding NUNC 96-well plate using a Perkin-Elmer (Enspire) multimode plate reader by varying the excitation wavelength (230–290 nm) and measuring emmision (300–500 nm) in steps of 3 nm in both dimensions. The data were plotted using the OriginPro 9.1 software (OriginLab Corporation, Northampton, MA, USA).

### Molecular modelling

The structure of **10a**, based on the X-ray crystal structure, was solvated in a 15-Å^3^ custom-built acetonitrile box, and the resulting system was minimized with the default presimulation protocol in Desmond (Desmond Molecular Dynamics System, version 3.4, D. E. Shaw Research, New York, NY, 2013; *Maestro-Desmond Interoperability Tools, version 3.4*, Schrödinger, 2013) consisting of (i) minimization with solute restraints, (ii) minimization without restraints, (iii) Berendsen NVT simulation at *T*=10 K with small time steps and restraints on heavy solute atoms, (iv) Berendsen NPT simulation at *T*=10 K with restraints on heavy solute atoms, (v) Berendsen NPT simulation with restraints on heavy solute atoms and (vi) unrestrained Berendsen NPT simulation^44^. Then, NPT simulations with periodic boundary conditions were carried out for 250 ns with the Nose–Hoover chain thermostat^45^,^46^ set to 300 K and a relaxation time of 1 ps. The Martyna–Tobias–Klein barostat^47^ was used to regulate pressure to 1 bar with isotropic coupling and a relaxation time of 2 ps. Equations of motion were integrated using the RESPA integrator^48^ with bonded, near and far, time steps of 2, 2 and 6 femtoseconds, respectively. Non-bonded interactions were subjected to a cutoff of 9 Å. Long-range electrostatics were treated with the smooth-particle mesh Ewald method^49^ with a tolerance of 10^−9^. The OPLS-2005 force field^50^,^51^ was used to describe both **10a** and solvent. All atomic coordinates and corresponding molecular mechanics energies were saved at 25-ps intervals for the entire 250-ns simulation. The structure obtained after the presimulation protocol for the reference simulation at 300 K was also used as an initial structure for additional 250-ns simulations carried out at 200, 250, 350 and 400 K to ease comparison between the different temperatures. Finally, a 1-μs simulation was carried out at 300 K as well. The molecular dynamics trajectories were analysed using visual molecular dynamics^52^.

## Author contributions

J.S.L. and C.A.O. designed the research, analyzed the data and wrote the manuscript with input from all authors. J.S.L. performed syntheses, NMR spectroscopy, CD spectroscopy, compound crystallization and fluorescence measurements. P.H. solved the X-ray crystal structures. P.F. performed the molecular dynamics calculations.

## Additional information

**Accession codes**: Coordinates and structure factors for the X-ray crystal structures of compounds **10a** (1054162) and compound **10c** (1054163), respectively, are deposited in the Cambridge Crystallographic Data Centre under the accession numbers mentioned above.

**How to cite this article:** Laursen, J.S. *et al.* Triangular prism-shaped β-peptoid helices as unique biomimetic scaffolds. *Nat. Commun.* 6:7013 doi: 10.1038/ncomms8013 (2015).

## Supplementary Material

Supplementary Figures, Supplementary Tables and Supplementary References Supplementary Figures 1-49, Supplementary Tables 1-4 and Supplementary References

Supplementary Movie 1 Molecular dynamics simulation of compound 10a in acetonitrile at 300 K.

Supplementary Data 1 Crystallographic data for compound 10c

Supplementary Data 2 Crystallographic data for compound 10a

## Figures and Tables

**Table 1:** Overall *cis–trans*-amide bond ratios.

**Table 2:** Torsion angles in the helical structures.
