# Supplementary material for: Triangular prism-shaped β-peptoid helices as unique biomimetic scaffolds
Source: Nat Commun. 2015 May 6;6:7013. doi: 10.1038/ncomms8013 (PMC4432622; doi:10.1038/ncomms8013)
Supplement: Supplementary Figures, Supplementary Tables and Supplementary References — Supplementary Figures 1-49, Supplementary Tables 1-4 and Supplementary References [file ncomms8013-s1.pdf]

Supplementary Figures

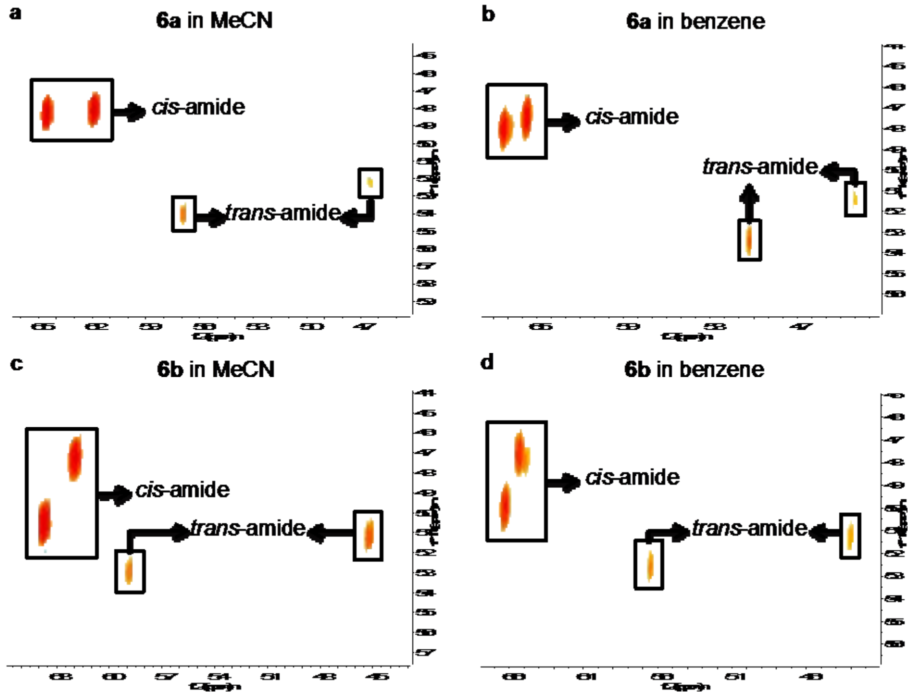

Supplementary Figure 1 | HSQC spectra. Spectra showing the side chain methine proton from (**a**) compound **6a** in MeCN-d_3, (**b**) compound **6a** in benzene-d_6, (**c**) compound **6b** in MeCN-d_3, and (**d**) compound **6b** in benzene-d_6.

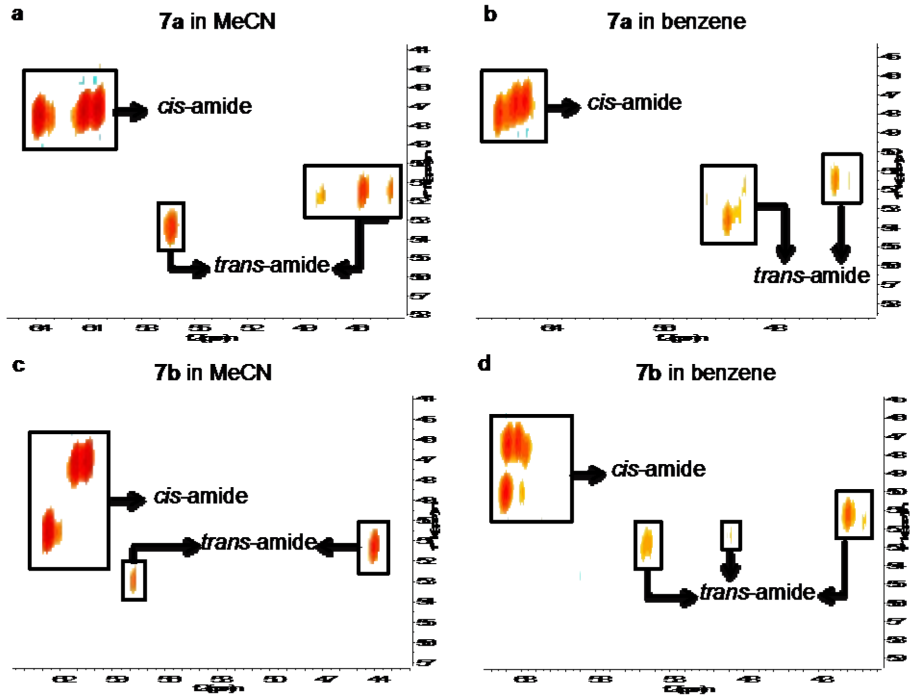

Supplementary Figure 2 | HSQC spectra. Spectra showing the side chain methine proton from (**a**) compound **7a** in MeCN-d_3, (**b**) compound **7a** in benzene-d_6, (**c**) compound **7b** in MeCN-d_3, and (**d**) compound **7b** in benzene-d_6.

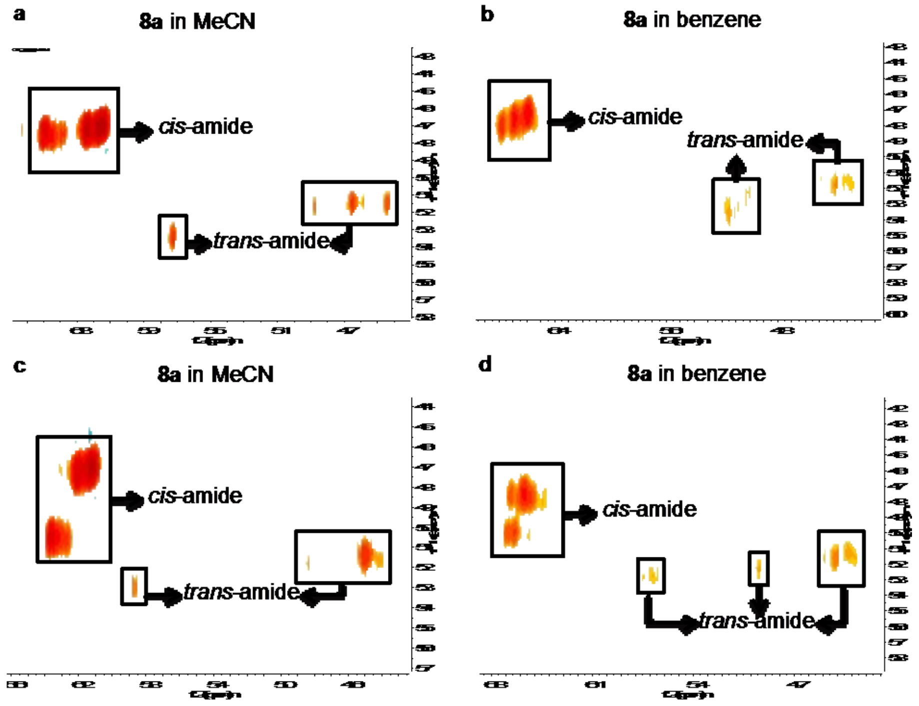

Supplementary Figure 3 | HSQC spectra. Spectra showing the side chain methine proton from (**a**) compound **8a** in MeCN-d_3, (**b**) compound **8a** in benzene-d_6, (**c**) compound **8b** in MeCN-d_3, and (**d**) compound **8b** in benzene-d_6.

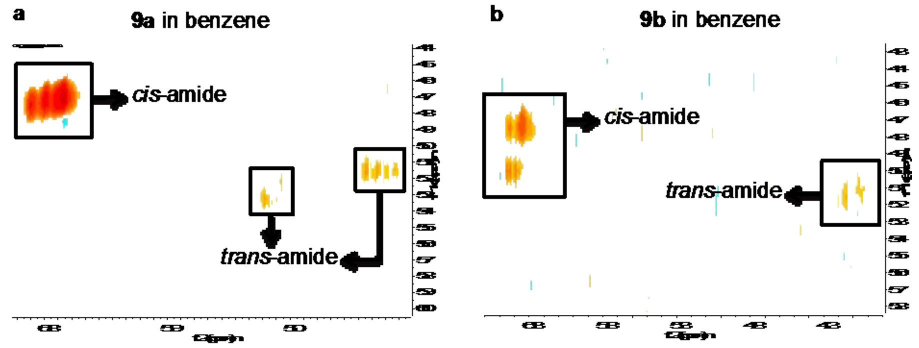

Supplementary Figure 4 | HSQC spectra. Spectra showing the side chain methine proton from (**a**) compound **9a** in MeCN-d_3, (**b**) compound **9a** in benzene-d_6, (**c**) compound **9b** in MeCN-d_3, and (**d**) compound **9b** in benzene-d_6.

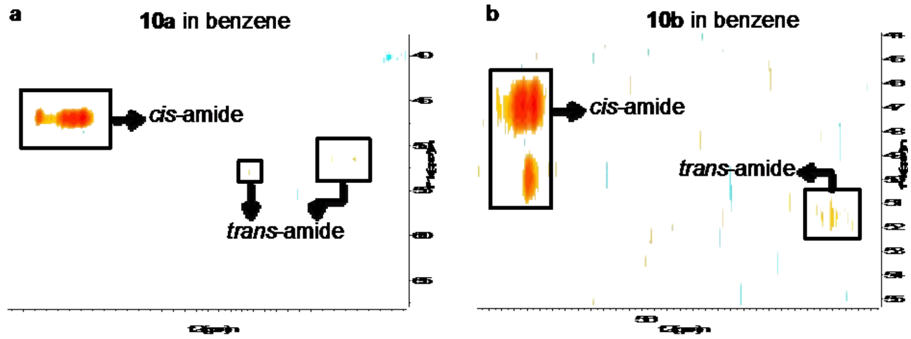

Supplementary Figure 5 | HSQC spectra. Spectra showing the side chain methine proton from (**a**) compound **10a** in MeCN-d_3, (**b**) compound **10a** in benzene-d_6, (**c**) compound **10b** in MeCN-d_3, and (**d**) compound **10b** in benzene-d_6.

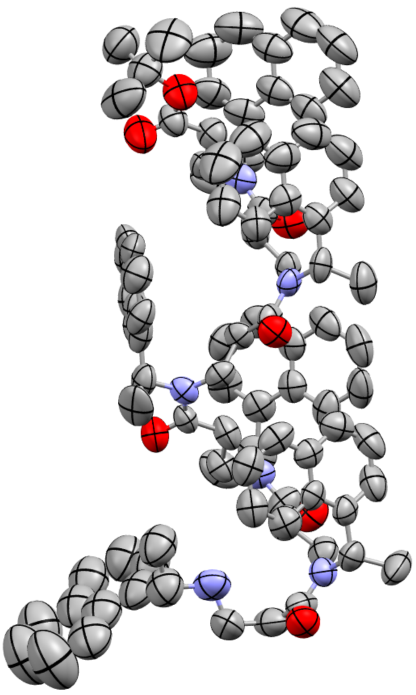

Supplementary Figure 6 | The asymmetric unit in crystal 10c. The 50% probability ellipsoid representaion was prepared using the Mercury software.[1]

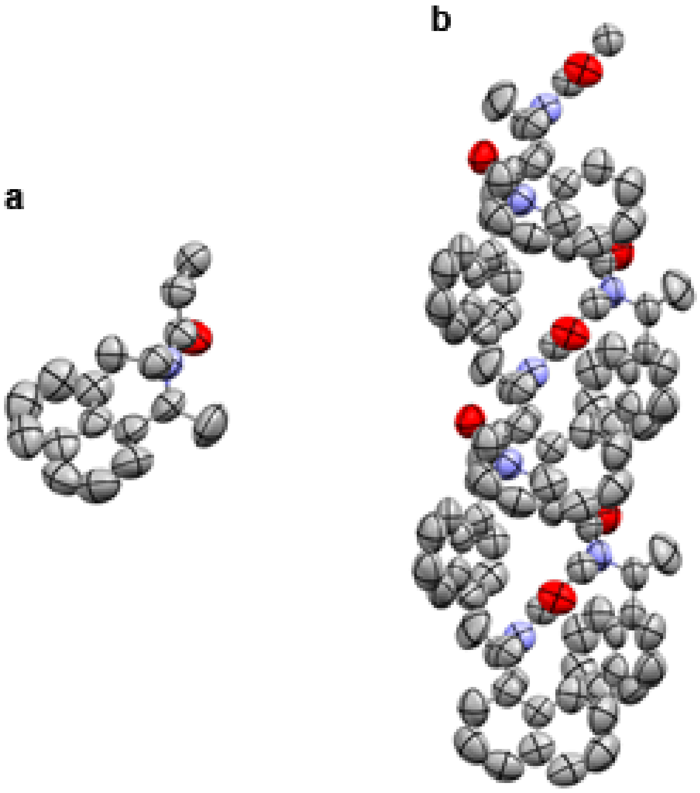

Supplementary Figure 7 | The asymmetric unit in crystal 10a. (**a**) The asymmetric unit. (**b**) The hexamer generated by imposing crystallographic symmetry. The 50% probability ellipsoid representaion was prepared using the Mercury software.[1]

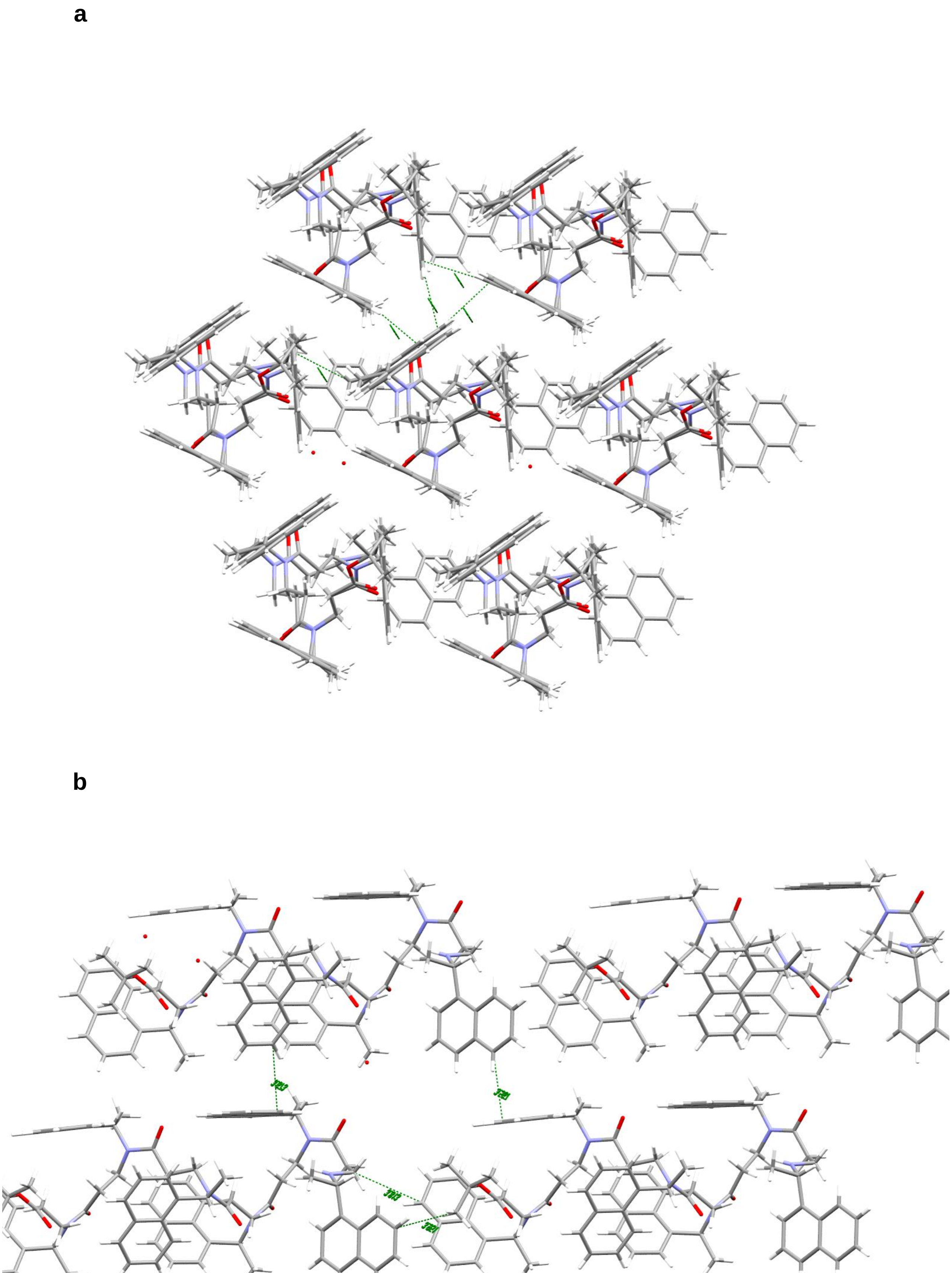

Supplementary Figure 8 | Crystal packing of 10c. (**a**) Viewed along the helical axis. The helices arrange in a pseudo-trigonal manner. (**b**) Perpendicular to the helical axis. The figure was prepared using the Mercury software.[1]

a

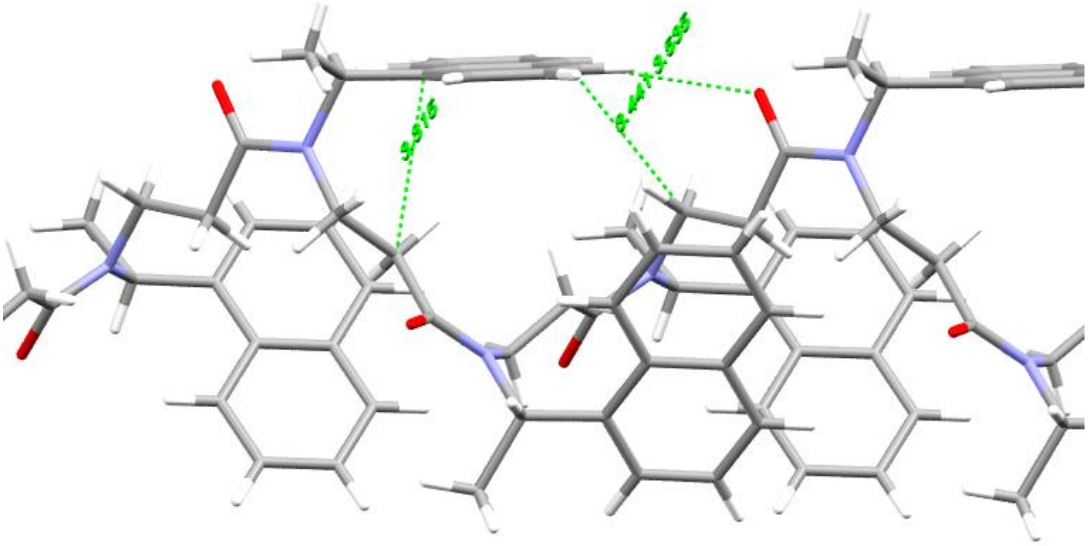

b

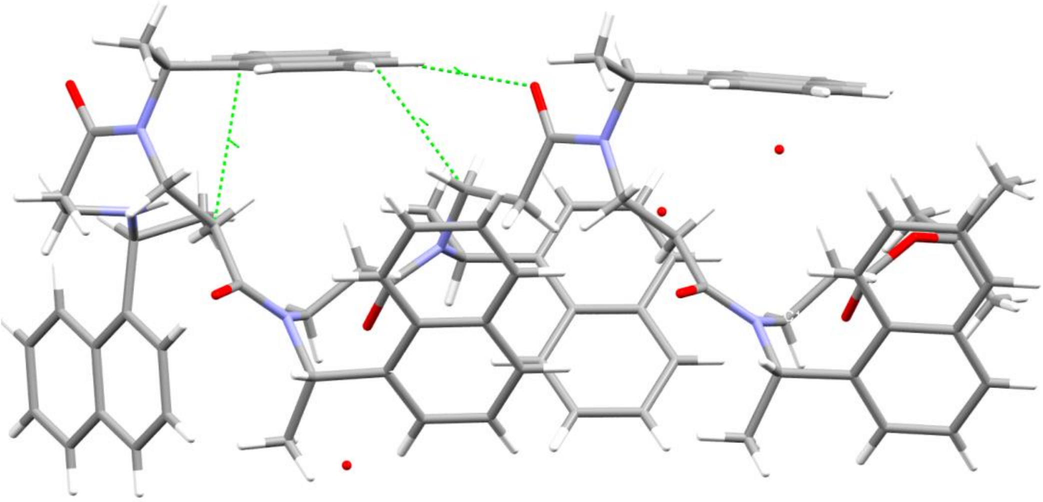

Supplementary Figure 9 | Backbone–side chain distances. (a) Naphthyl–backbone interactions in compound **10a**. (**b**) Naphthyl–backbone interactions in compound **10c**.

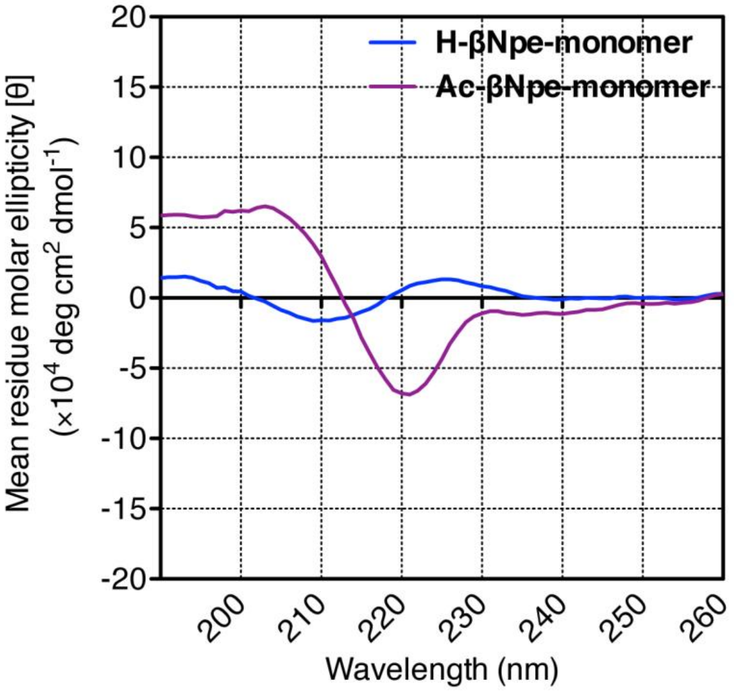

Supplementary Figure 10 | CD spectra. Spectra obtained for the acylated and non-acylated *(S)*-1-(1-naphthyl)ethyl-containing monomers in acetonitrile (60 μM) at room temperature.

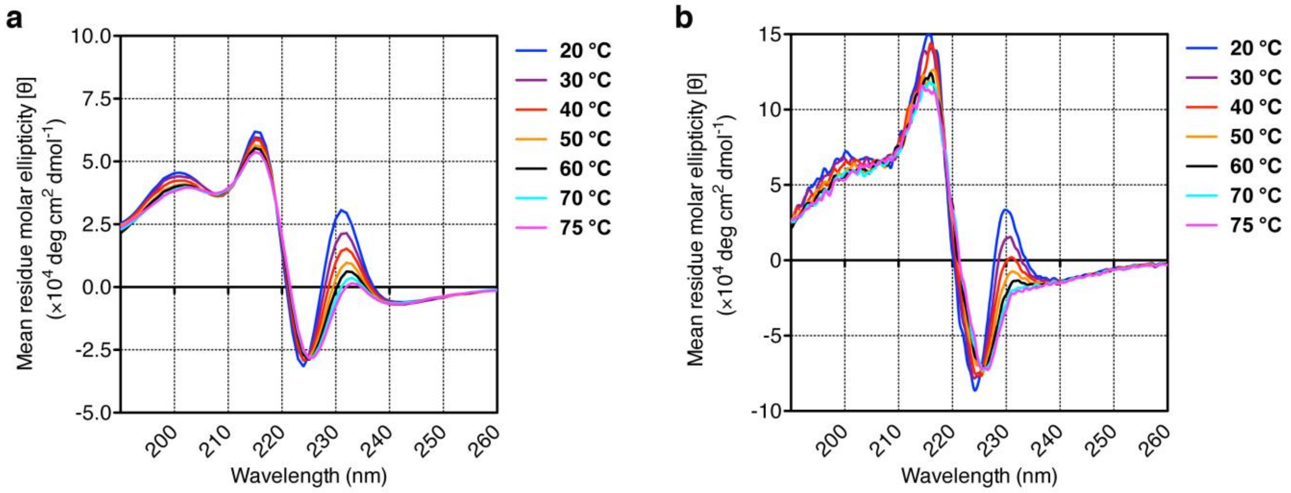

Supplementary Figure 11 | CD spectra. (**a**) Compound **10a** and (**b**) compound **10b** at varying temperatures in acetonitrile (~60 μM).

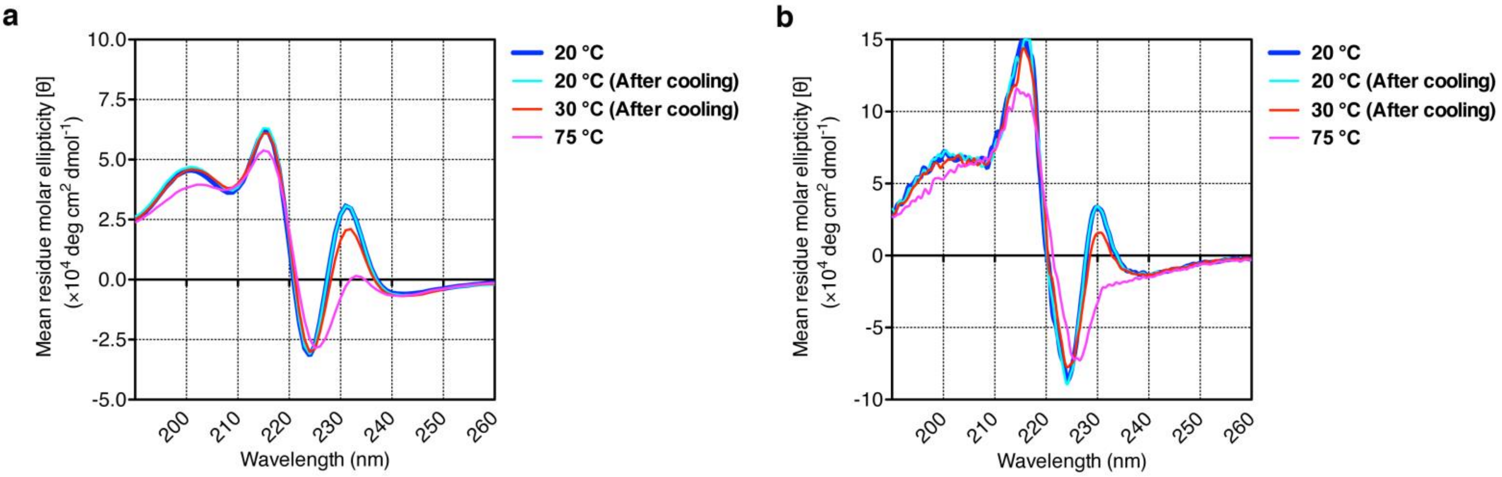

Supplementary Figure 12 | CD spectra. (**a**) Compound **10a** and (**b**) compound **10b** at varying temperatures in acetonitrile (~60 μM) upon cooling the sample back down to room temperature.

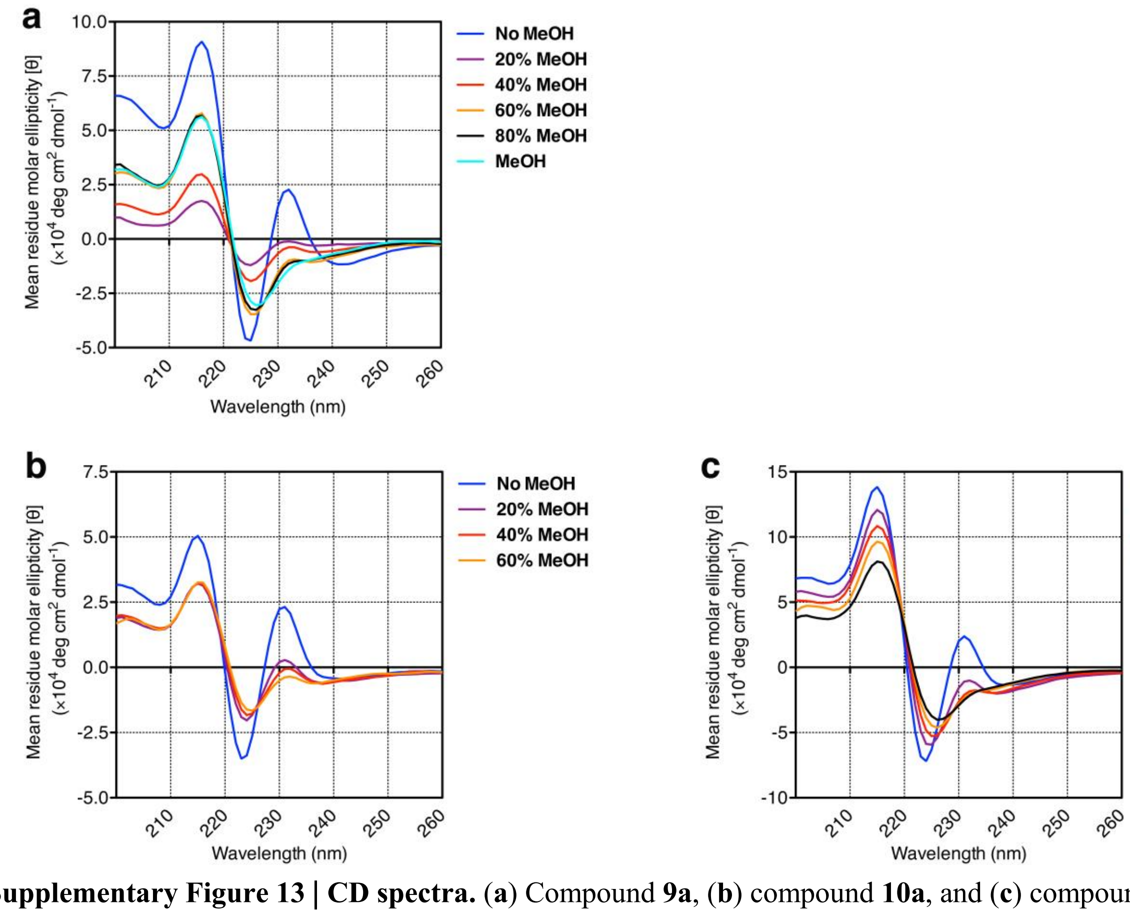

Supplementary Figure 13 | CD spectra. (**a**) Compound **9a**, (**b**) compound **10a**, and (**c**) compound **10b** recorded at varying concentrations of methanol (60 μM solutions). For the hexamers, poor solubility precluded spectra from being obtained at the highest concentrations of MeOH.

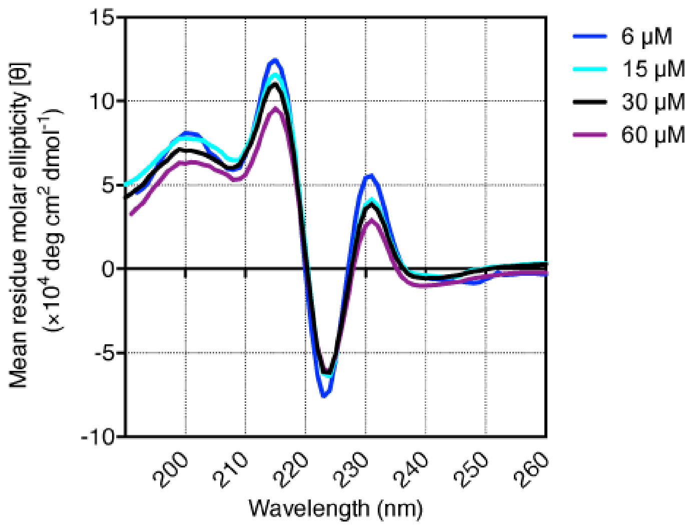

Supplementary Figure 14 | CD spectra. Compound **10a** recorded at varying concentrations in acetonitrile (60 μM solutions).

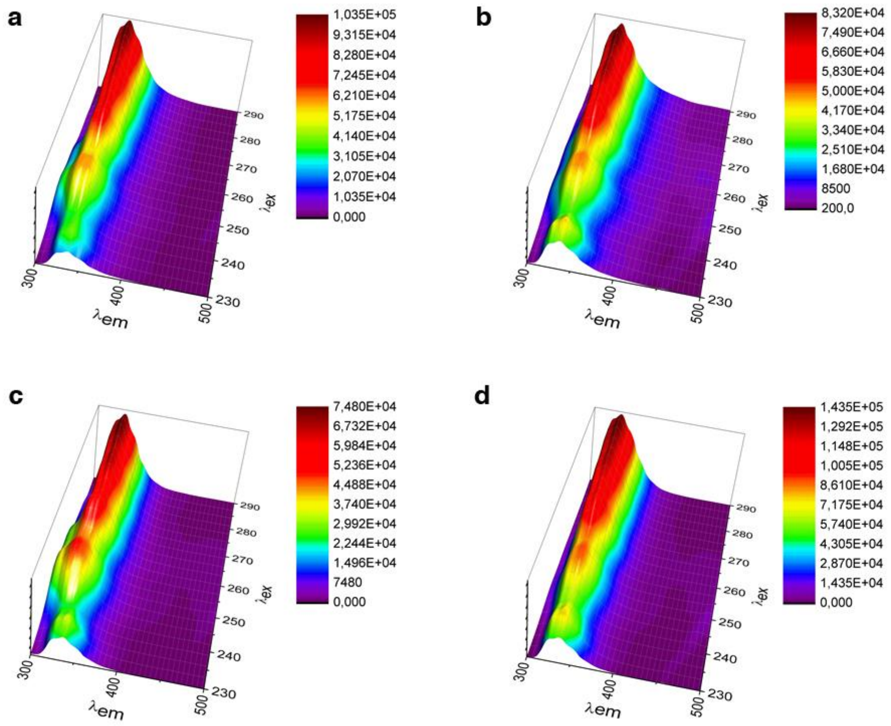

Supplementary Figure 15 | Fluorescence emission spectra. (**a**) Spectrum recorded for compound **10a** in acetonitrile or (**b**) in MeOH–acetonitrile (3:2). (**c**) Spectrum recorded for and compound **6a** in acetonitrile or (**d**) in MeOH–acetonitrile (3:2) at room temperature (60 μM solutions). The intensities are shown in arbitrary units (a.u.).

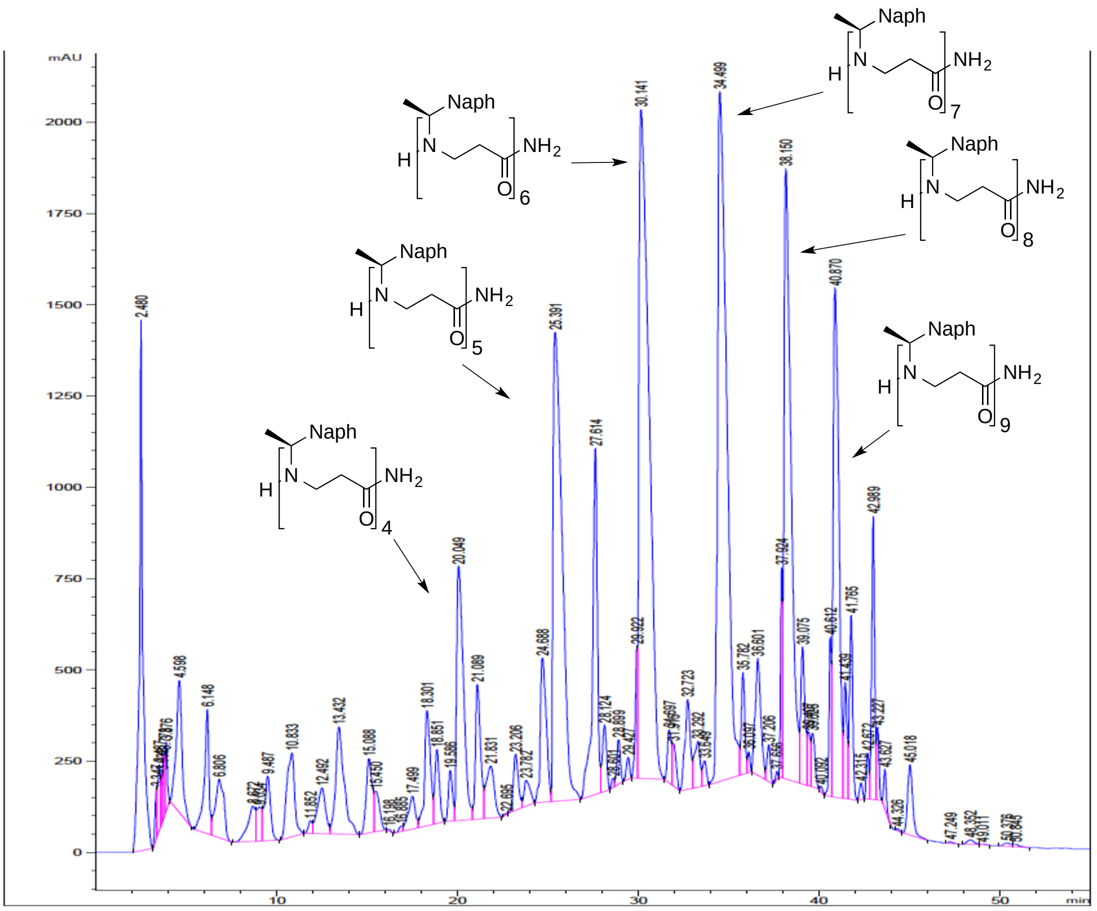

Supplementary Figure 16 | UV chromatogram of crude mixture from solid-phase synthesis. The chromatogram was obtained after sub-unit solid-phase synthesis aimed at compound **10e**. Compounds of four and five residues were identified by UPLC-MS, but were not collected during preparative HPLC purification.

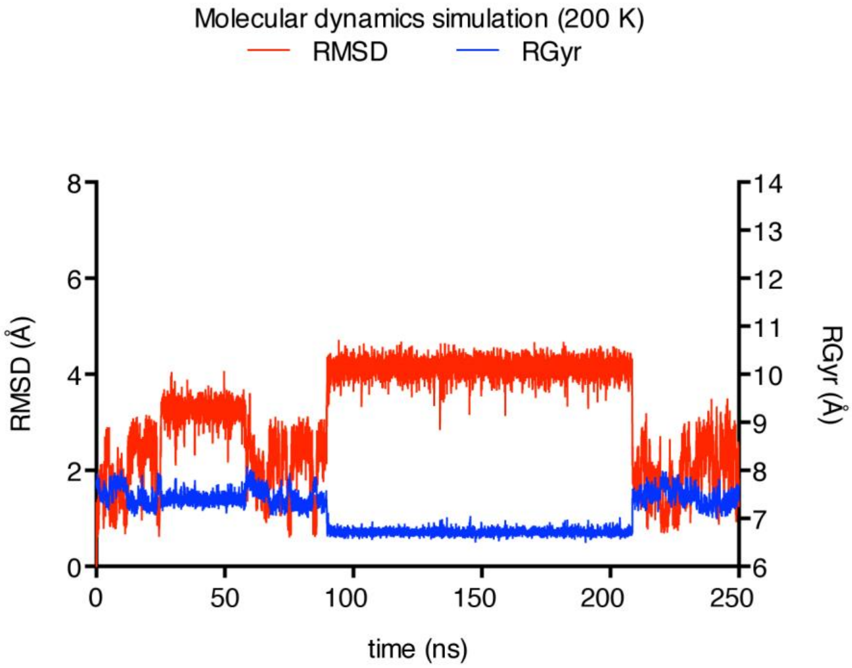

Supplementary Figure 17 | Molecular dynamics simulation. Root-mean square deviation (RMSD) and gyrational radius (RGyr) during MD simulation (250 ns) of compound **10a** at 200 K.

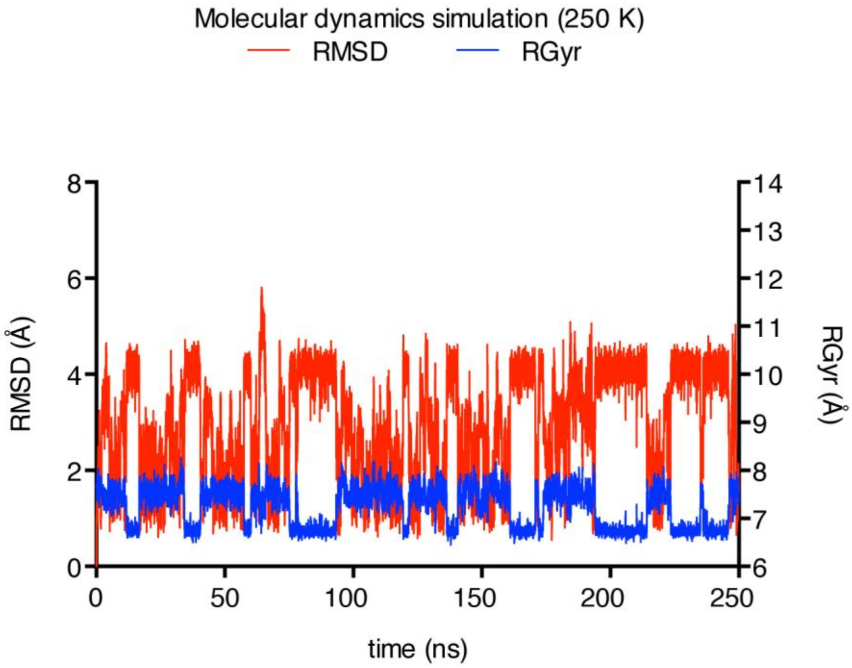

Supplementary Figure 18 | Molecular dynamics simulation. Root-mean square deviation (RMSD) and gyrational radius (RGyr) during MD simulation (250 ns) of compound **10a** at 250 K.

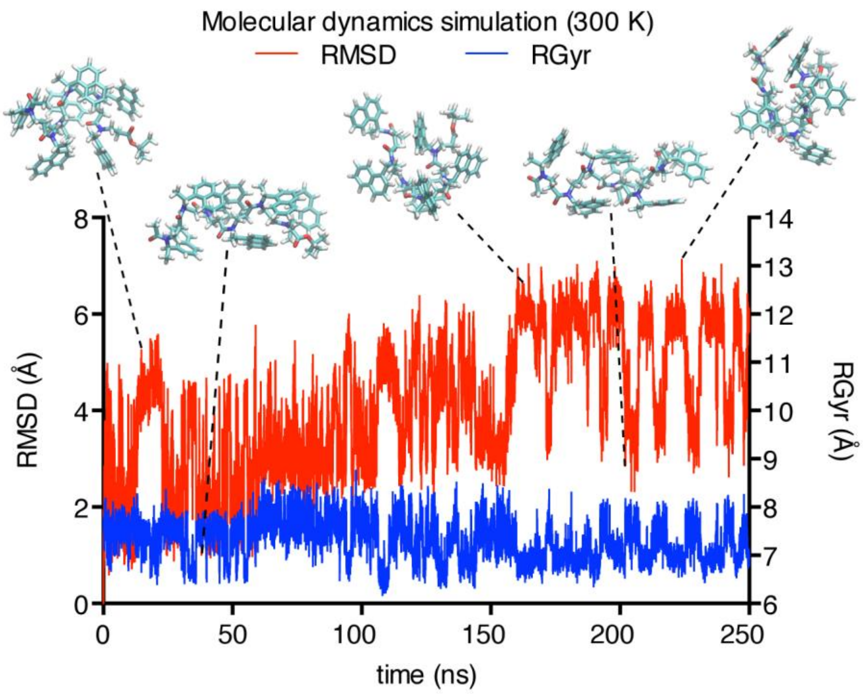

Supplementary Figure 19 | Molecular dynamics simulation. Root-mean square deviation (RMSD) and gyrational radius (RGyr) during MD simulation (250 ns) of compound **10a** at 300 K. Representative conformations are shown to illustrate the folded and unfolded states of the helical structure throughout the simulation.

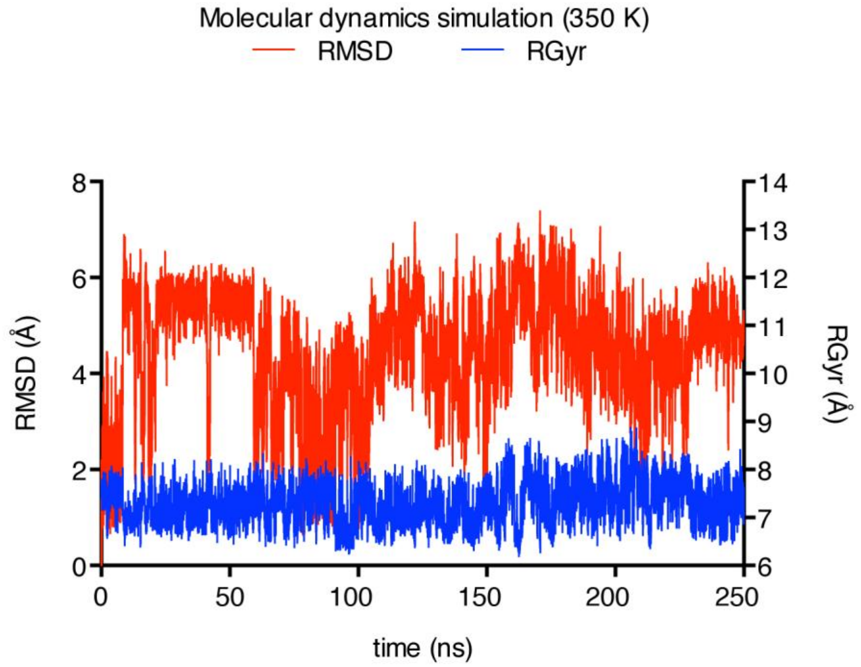

Supplementary Figure 20 | Molecular dynamics simulation. Root-mean square deviation (RMSD) and gyrational radius (RGyr) during MD simulation (250 ns) of compound **10a** at 350 K.

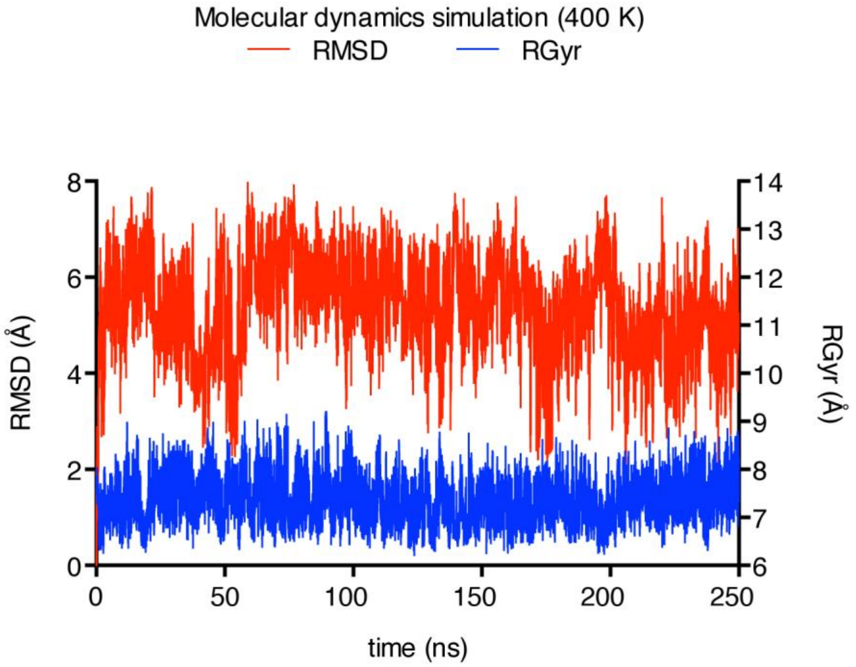

Supplementary Figure 21 | Molecular dynamics simulation. Root-mean square deviation (RMSD) and gyrational radius (RGyr) during MD simulation (250 ns) of compound **10a** at 400 K.

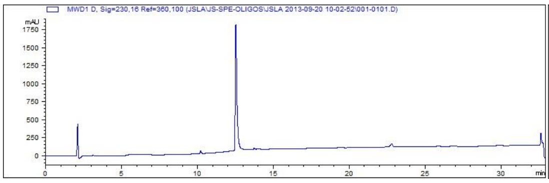

Compound **1a**

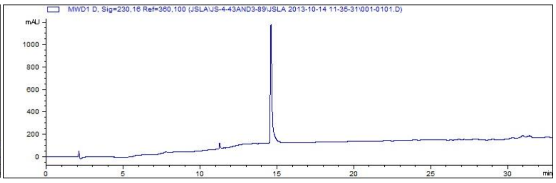

Com pound **1b**

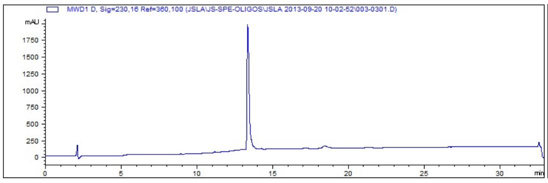

Compound **2a**

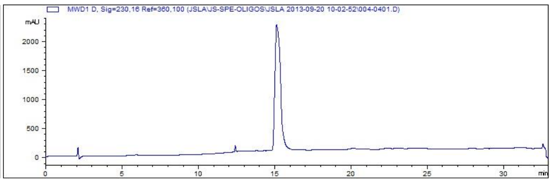

Com pound **2b**

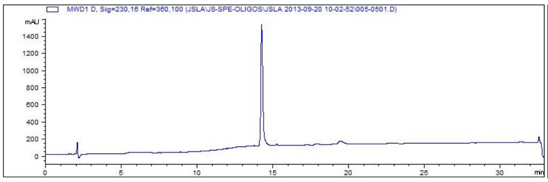

Compound **3a**

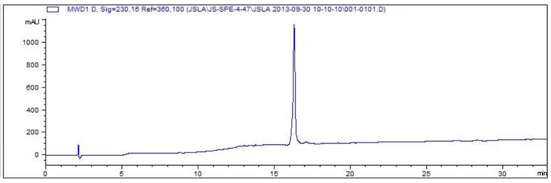

Com pound **3b**

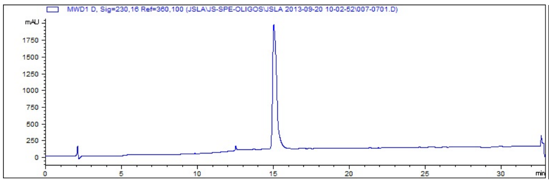

Compound **4a**

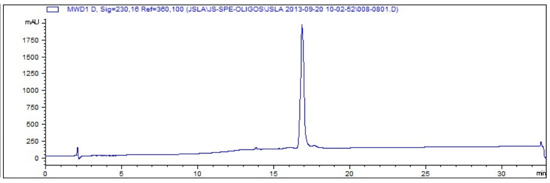

Com pound **4b**

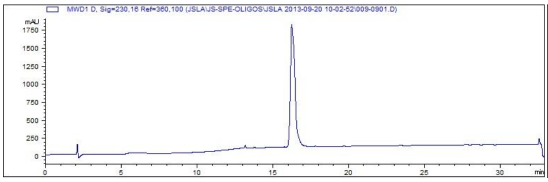

Compound **5a**

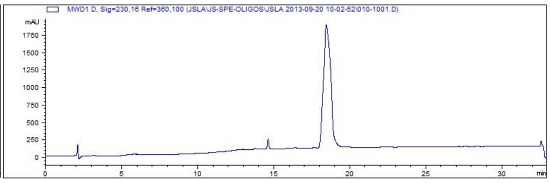

Com pound **5b**

Supplementary Figure 22 | HPLC traces of compounds 1a–5b.

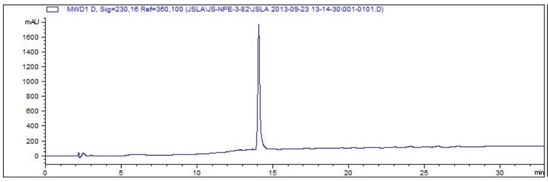

Compound **6a**

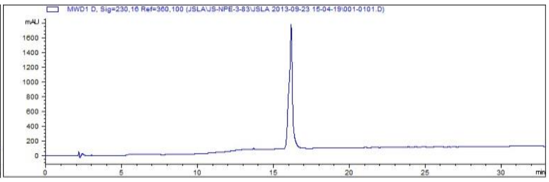

Com pound **6b**

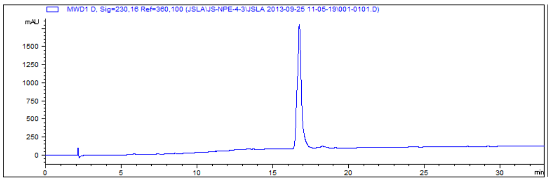

Compound **7a**

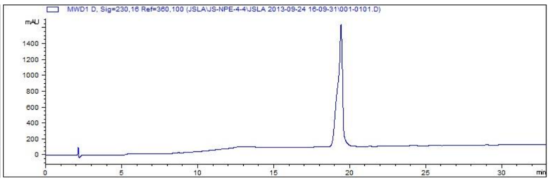

Com pound **7b**

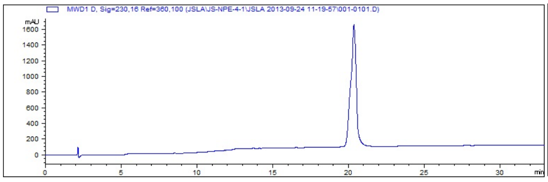

Compound **8a**

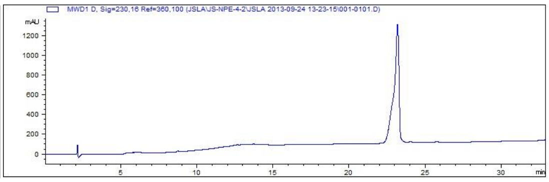

Com pound **8b**

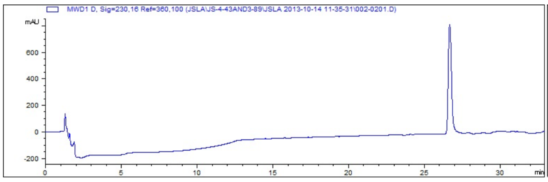

Compound **9a**

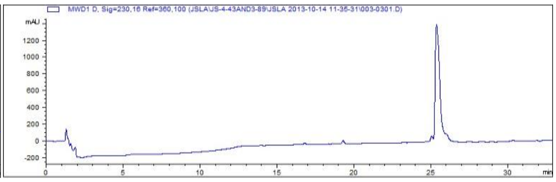

Com pound **9b**

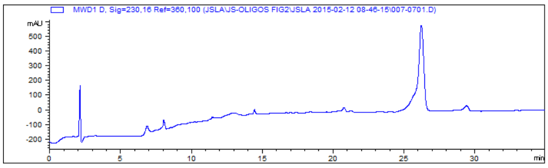

Compound **10a**

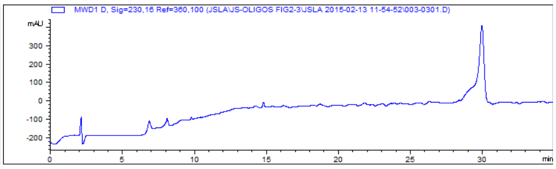

Com pound **10b**

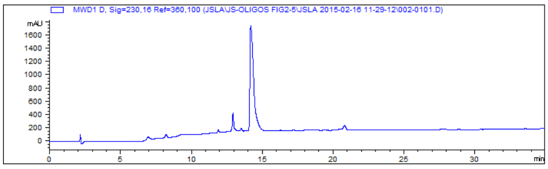

Compound **10c**

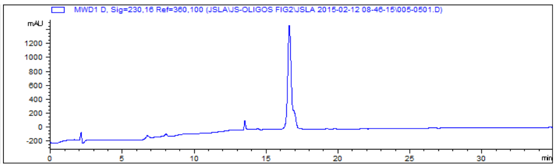

Com pound **10d**

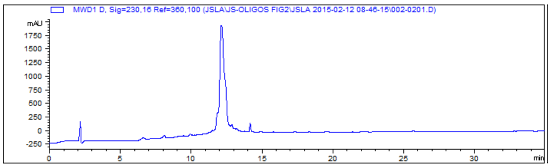

Compound **10e**

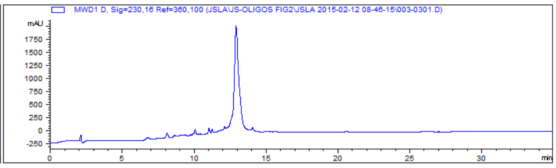

Com pound **11**

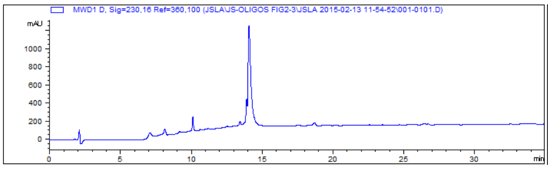

Compound **12**

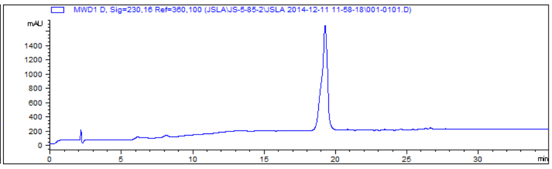

Com pound **16**

Supplementary Figure 23 | HPLC traces of compounds 6a–16.

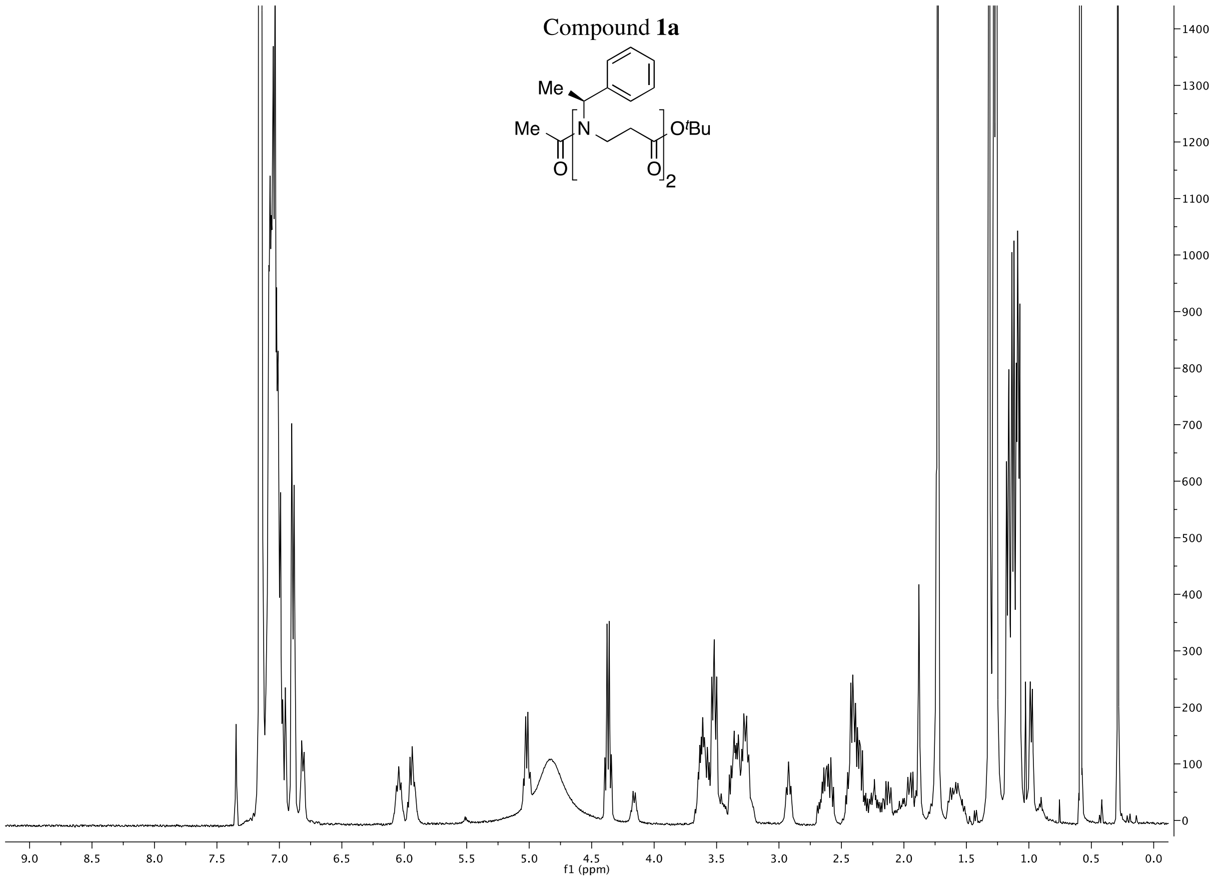

Supplementary Figure 24 | ^{1}H NMR spectrum of compound 1a. The spectrum was recorded in benzene-d_6 at room temperature.

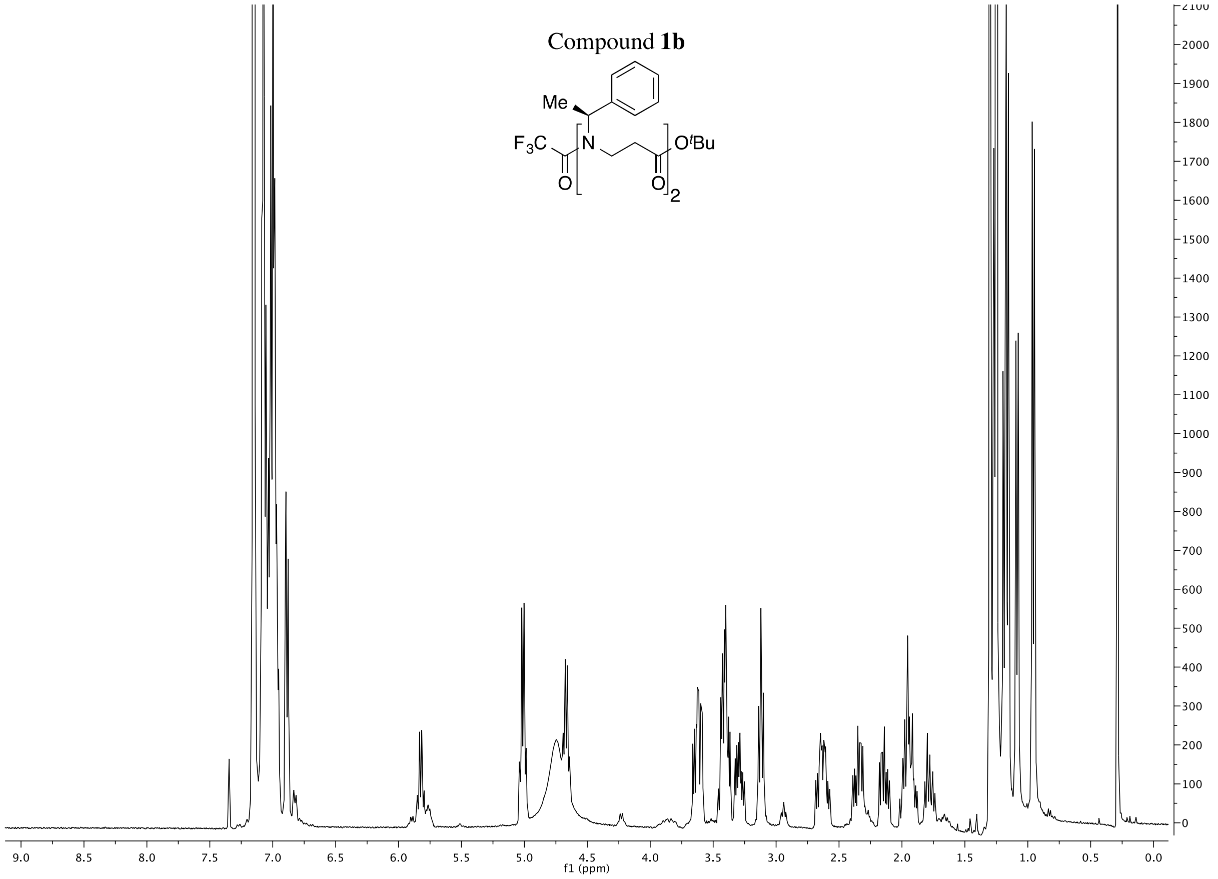

Supplementary Figure 25 | ^{1}H NMR spectrum of compound 1b. The spectrum was recorded in benzene-d_6 at room temperature.

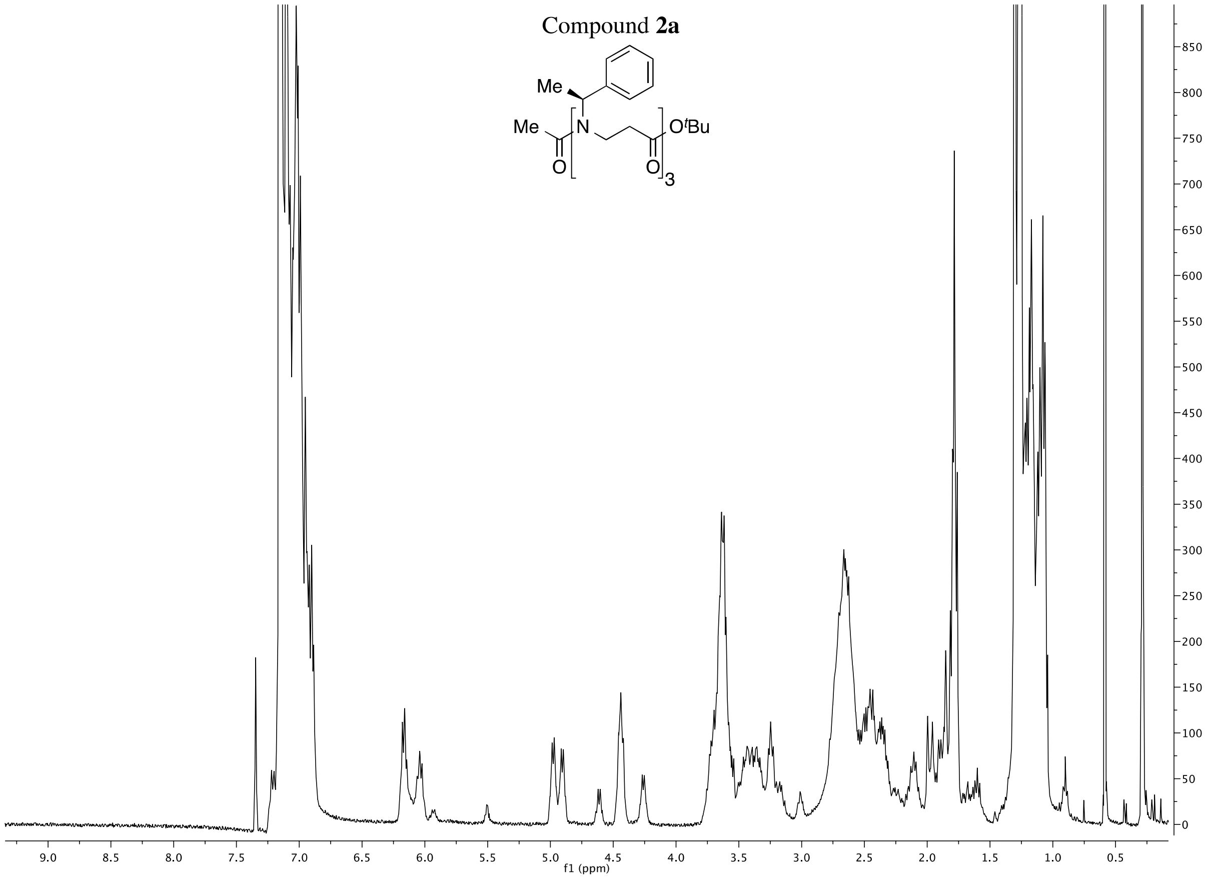

Supplementary Figure 26 | ^{1}H NMR spectrum of compound 2a. The spectrum was recorded in benzene-d_6 at room temperature.

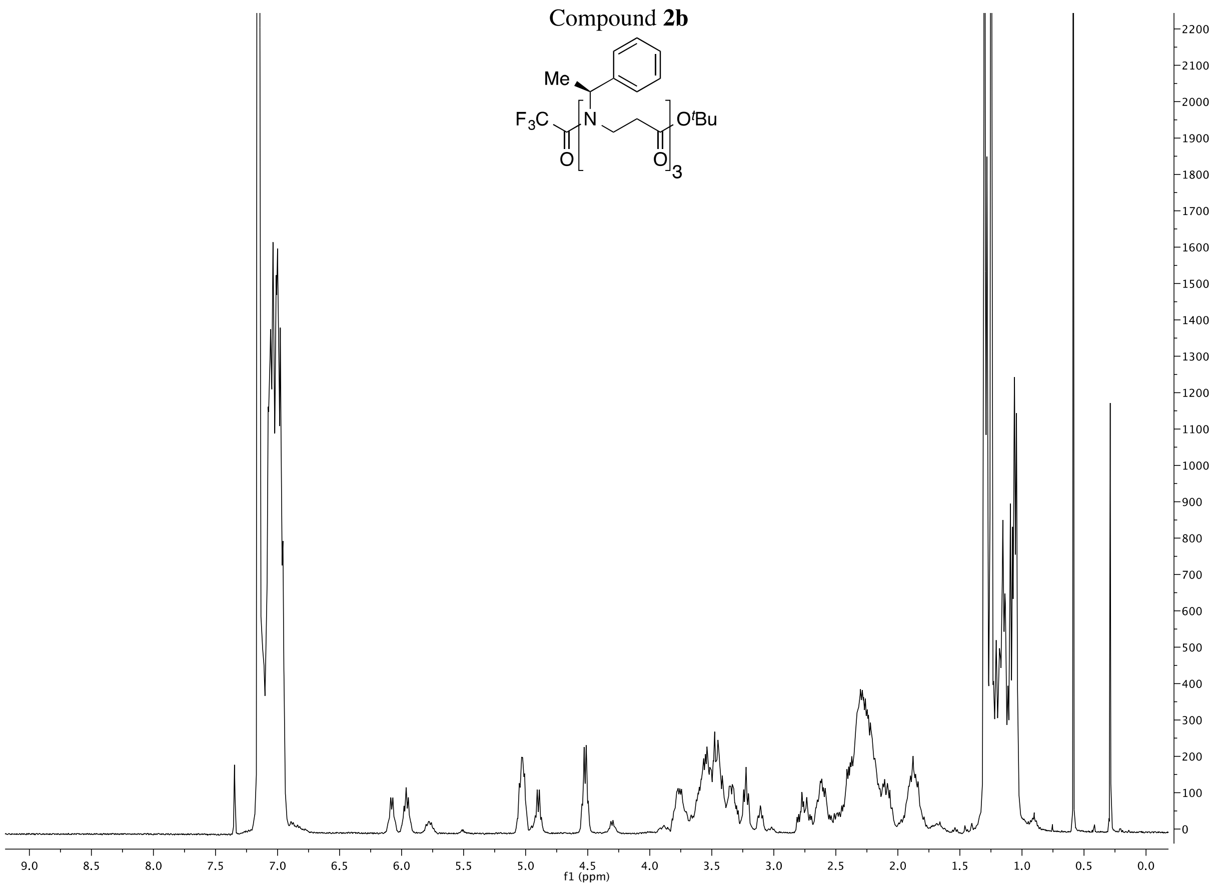

Supplementary Figure 27 | ^{1}H NMR spectrum of compound 2b. The spectrum was recorded in benzene-d_6 at room temperature.

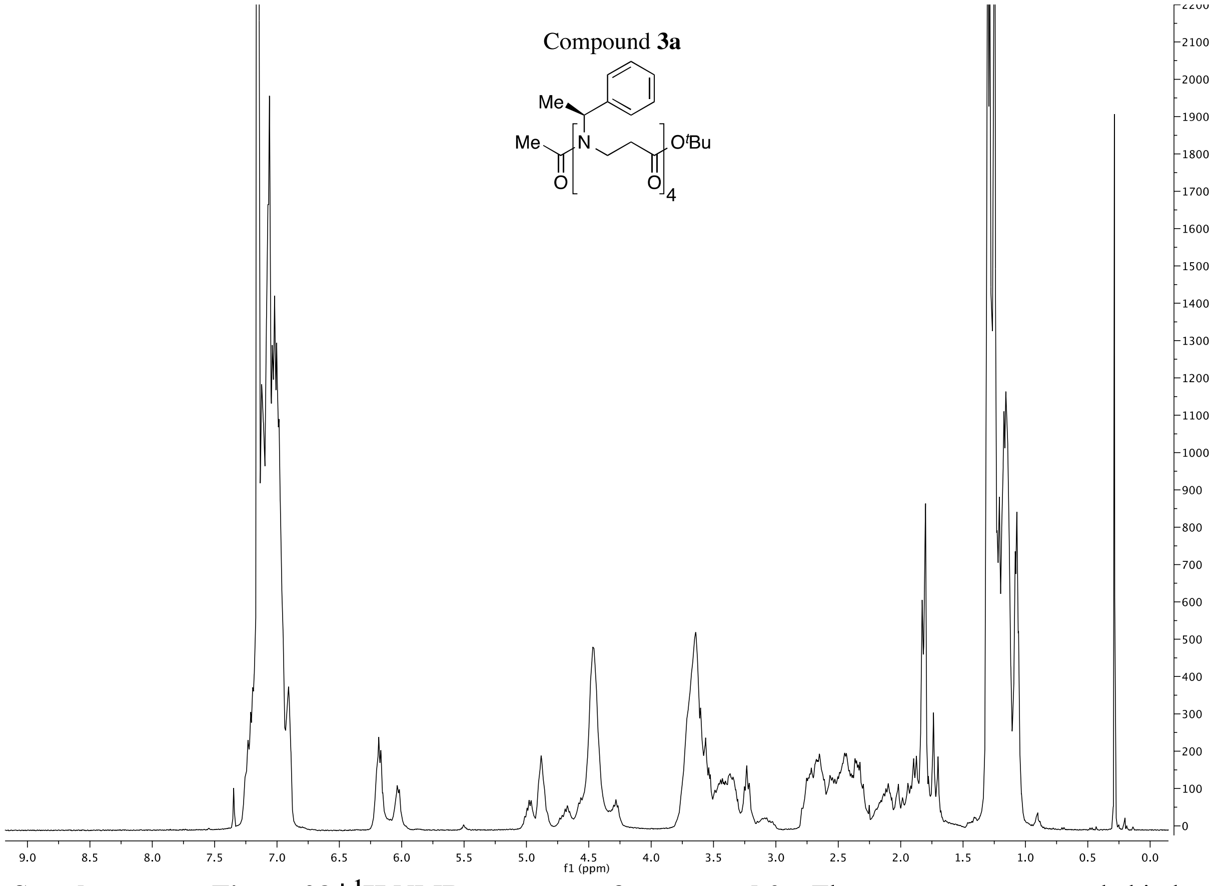

Supplementary Figure 28 | ^{1}H NMR spectrum of compound 3a. The spectrum was recorded in benzene-d_6 at room temperature.

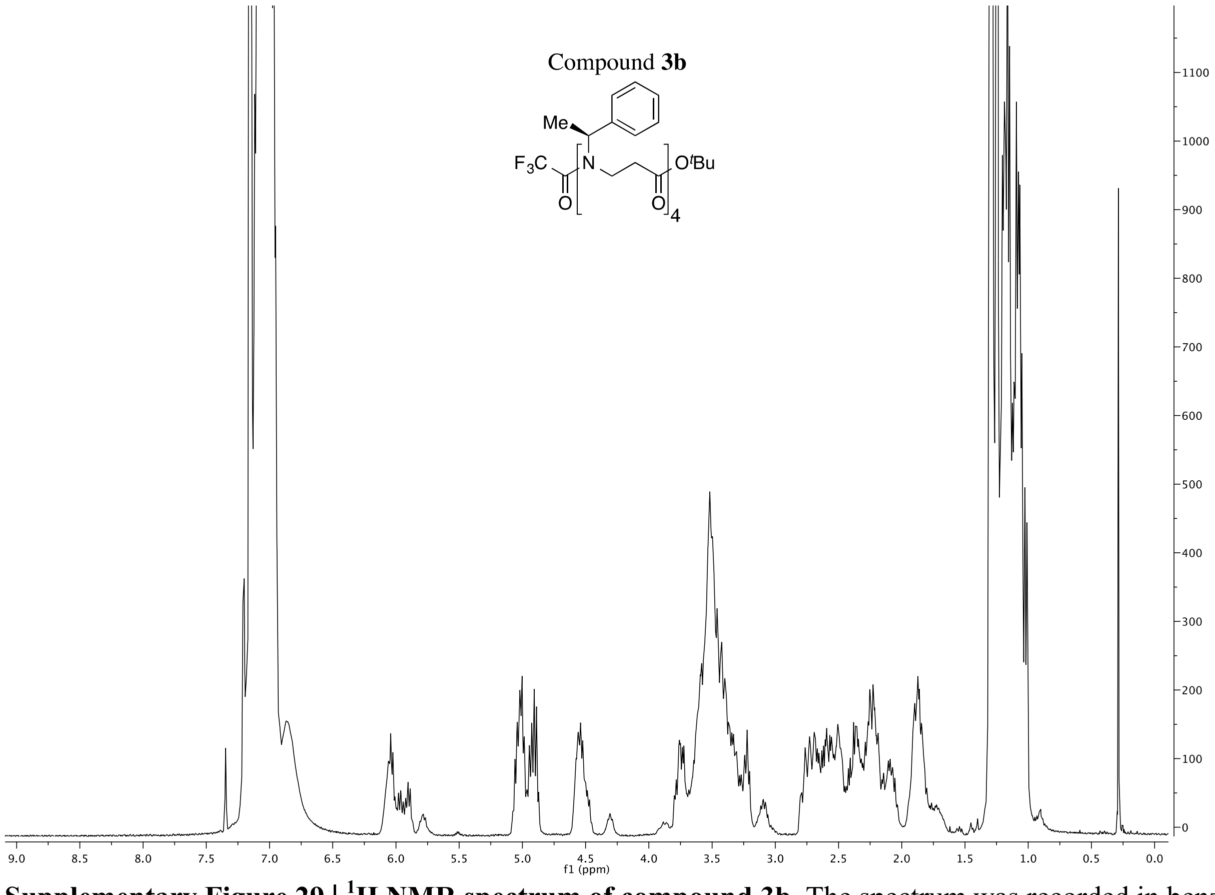

Supplementary Figure 29 | ^{1}H NMR spectrum of compound 3b. The spectrum was recorded in benzene-d_6 at room temperature.

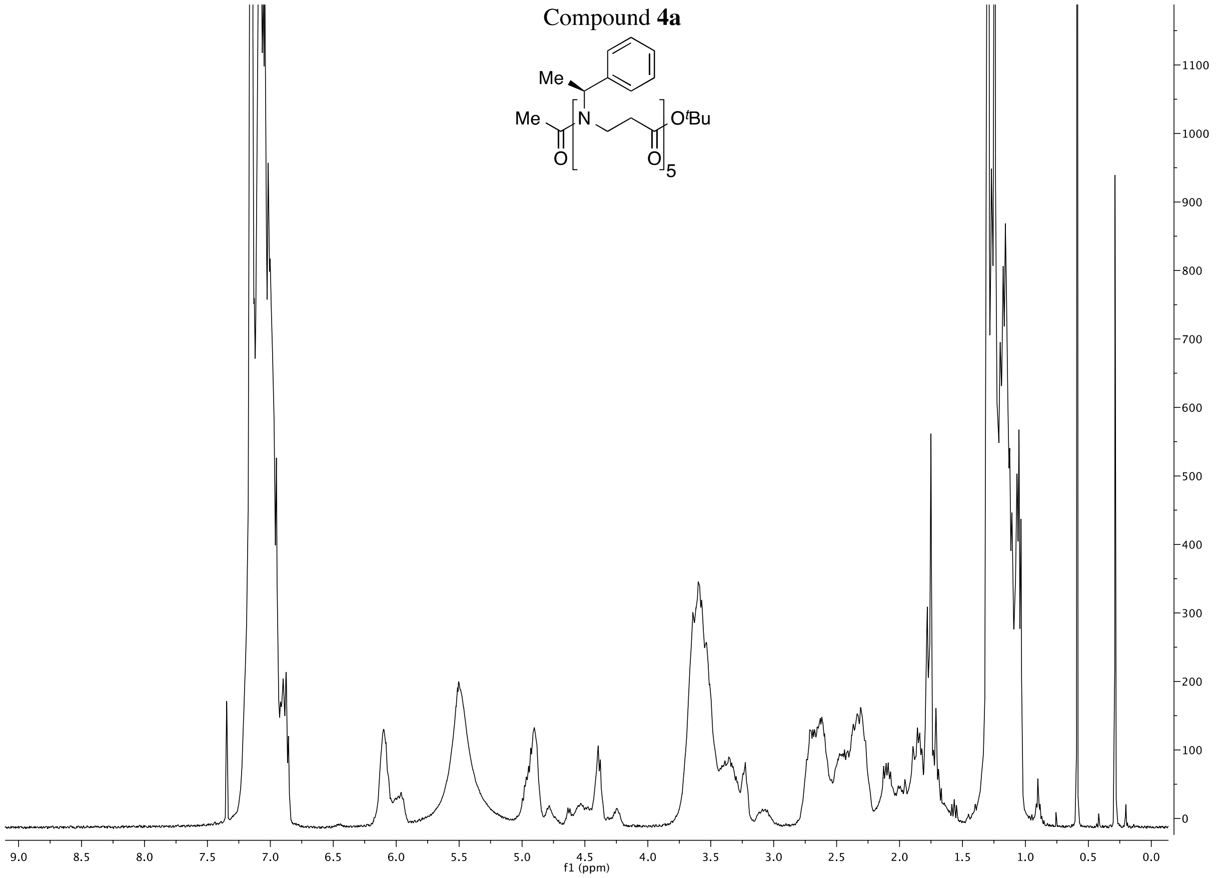

Supplementary Figure 30 | ^{1}H NMR spectrum of compound 4a. The spectrum was recorded in benzene-d_6 at room temperature.

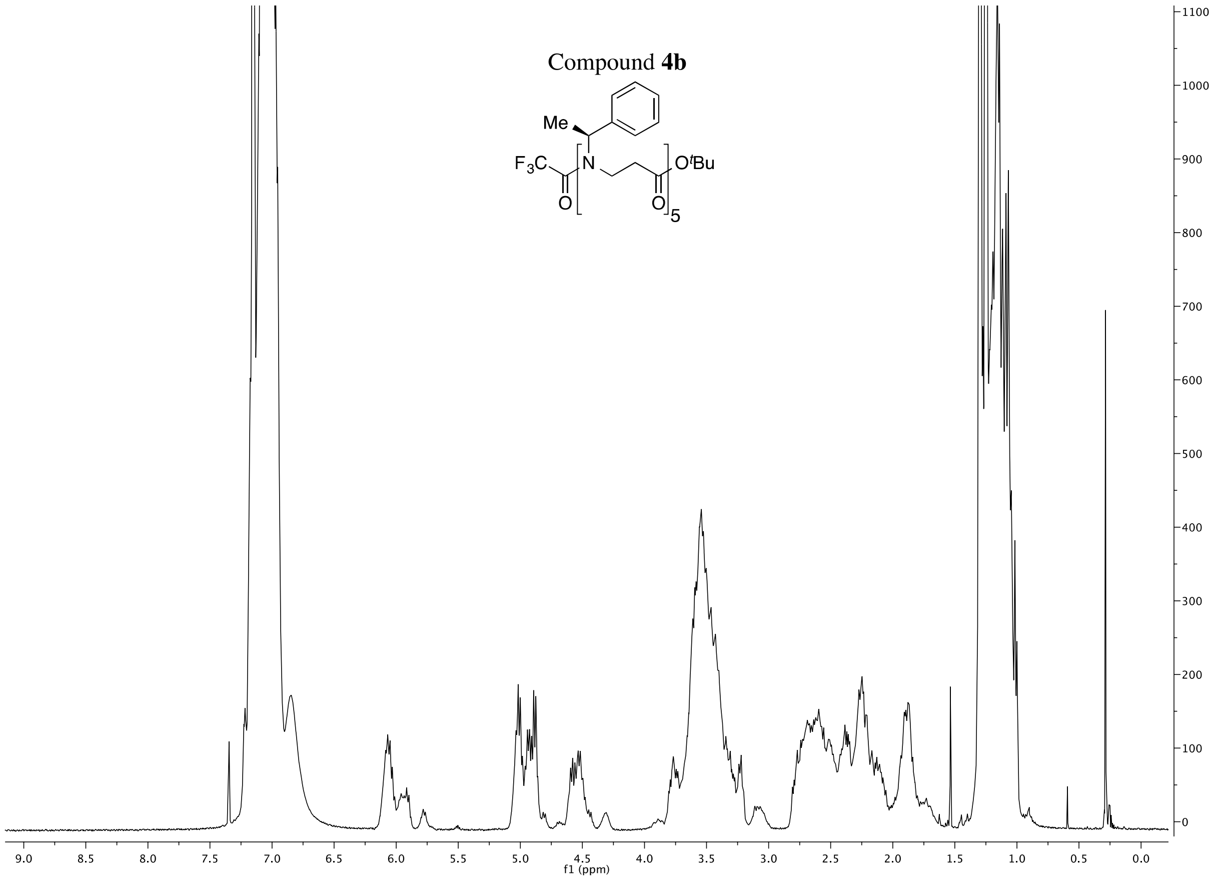

Supplementary Figure 31 | ^{1}H NMR spectrum of compound 4b. The spectrum was recorded in benzene-d_6 at room temperature.

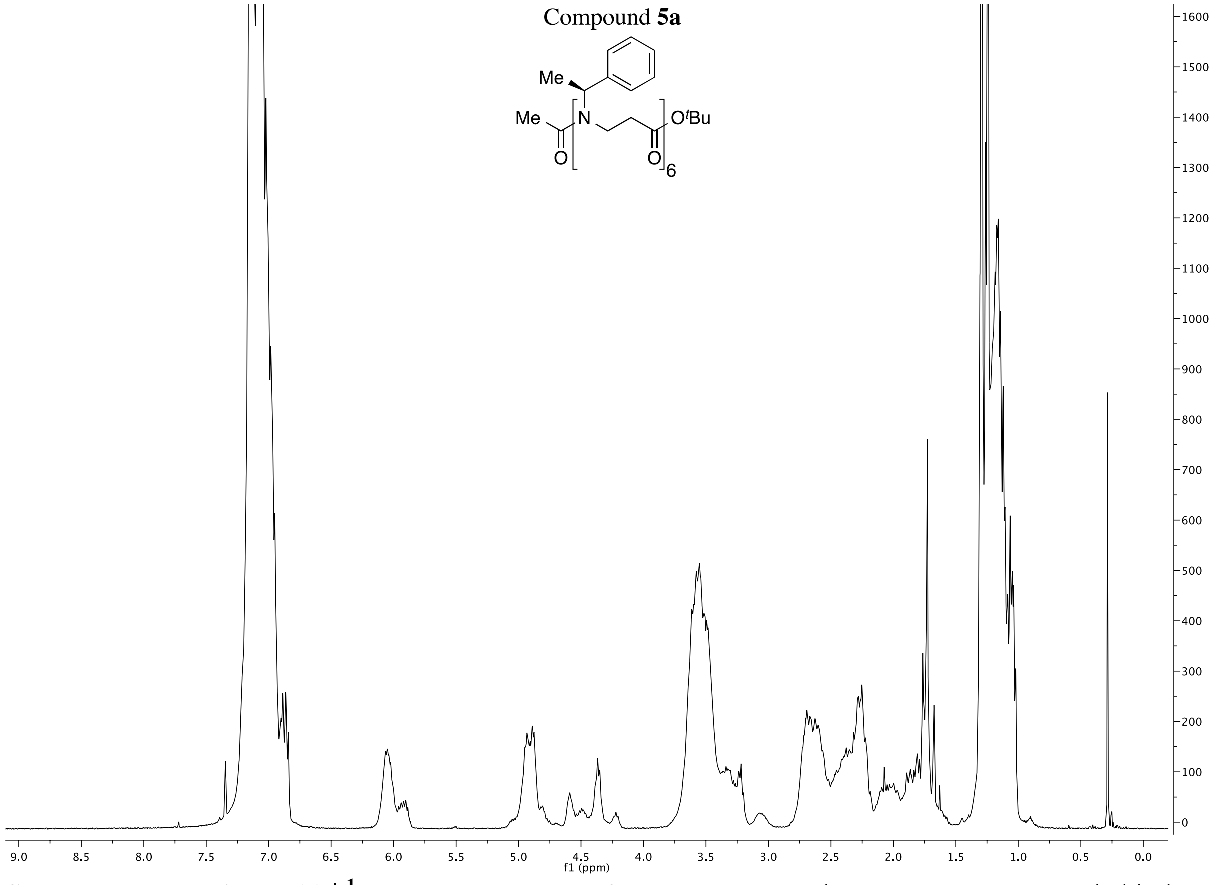

Supplementary Figure 32 | ^{1}H NMR spectrum of compound 5a. The spectrum was recorded in benzene-d_6 at room temperature

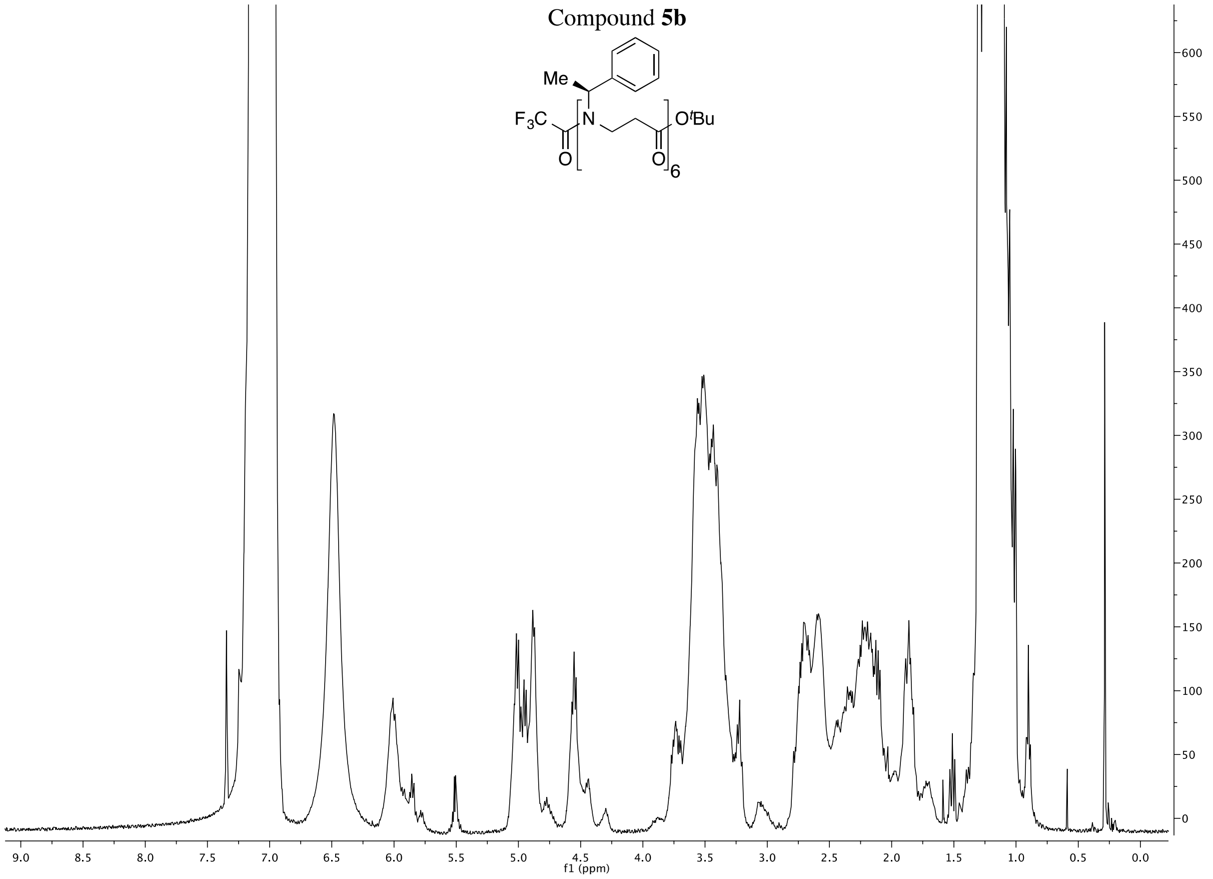

Supplementary Figure 33 | ^{1}H NMR spectrum of compound 5b. The spectrum was recorded in benzene-d_6 at room temperature.

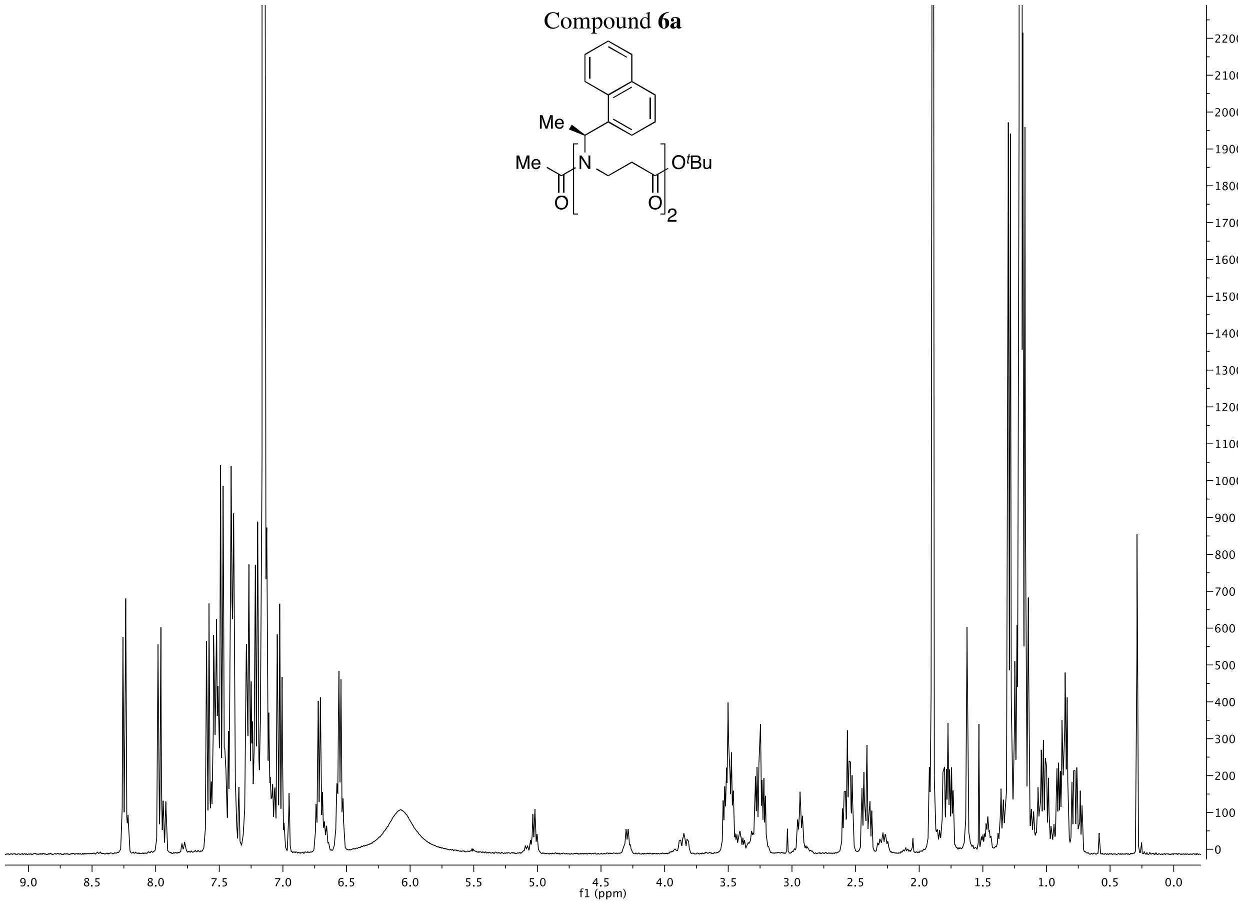

Supplementary Figure 34 | ^{1}H NMR spectrum of compound 6a. The spectrum was recorded in benzene-d_6 at room temperature.

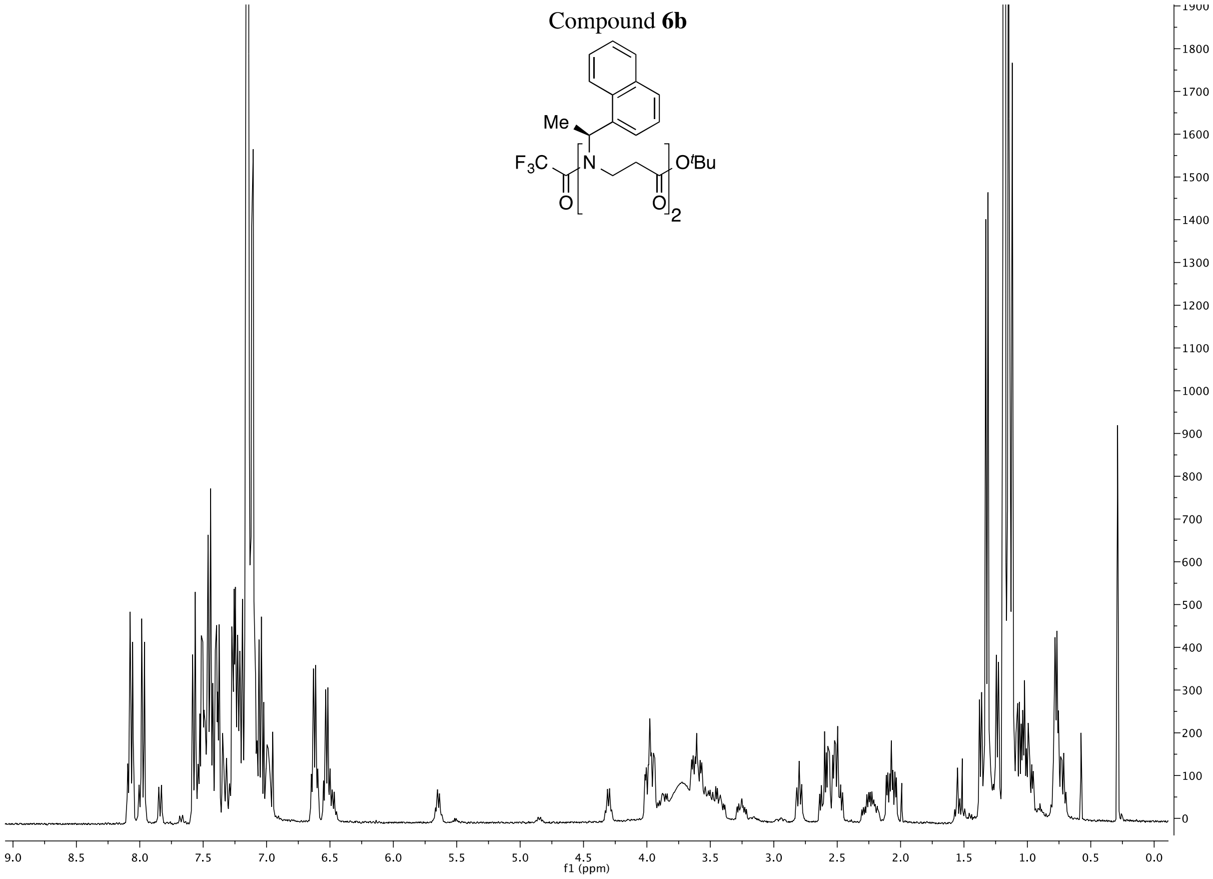

Supplementary Figure 35 | ^{1}H NMR spectrum of compound 6b. The spectrum was recorded in benzene-d_6 at room temperature.

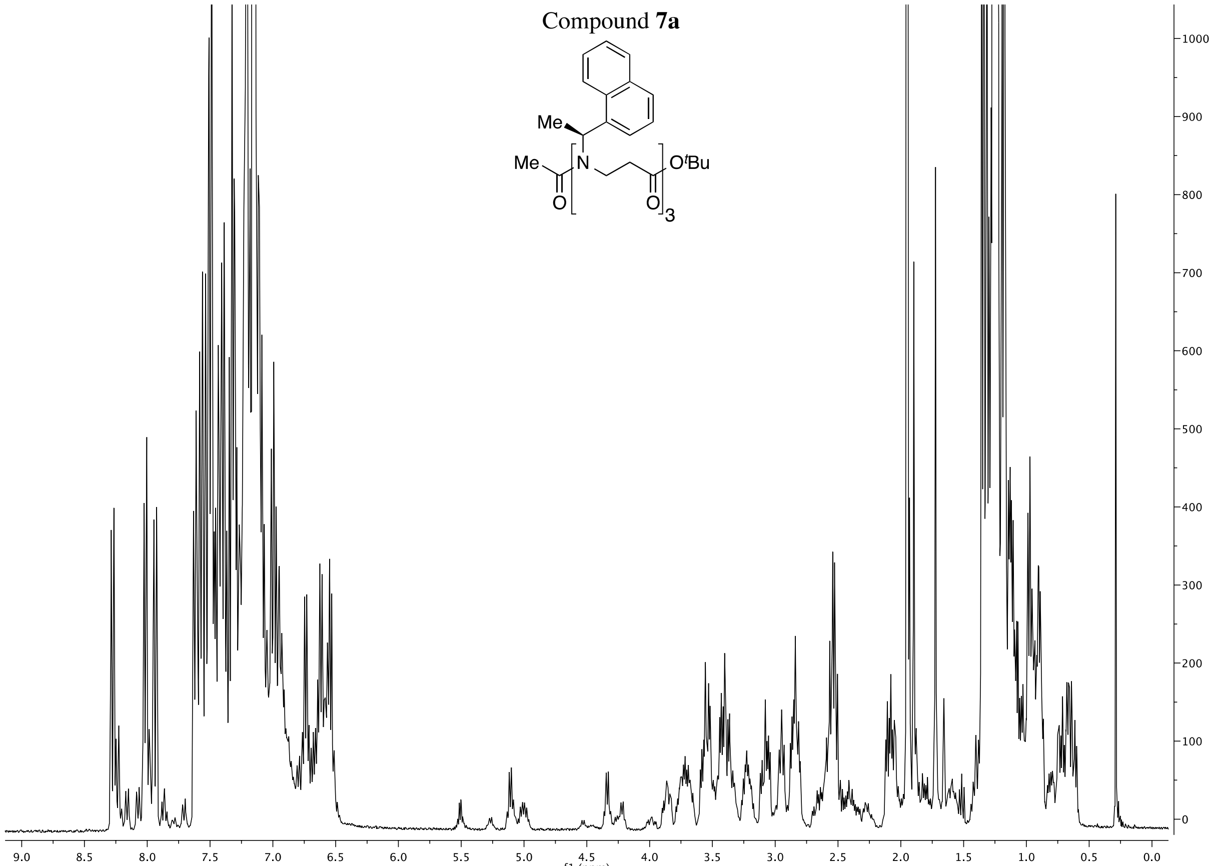

Supplementary Figure 36 | ¹H NMR spectrum of compound 7a. The spectrum was recorded in benzene-d_6 at room temperature.

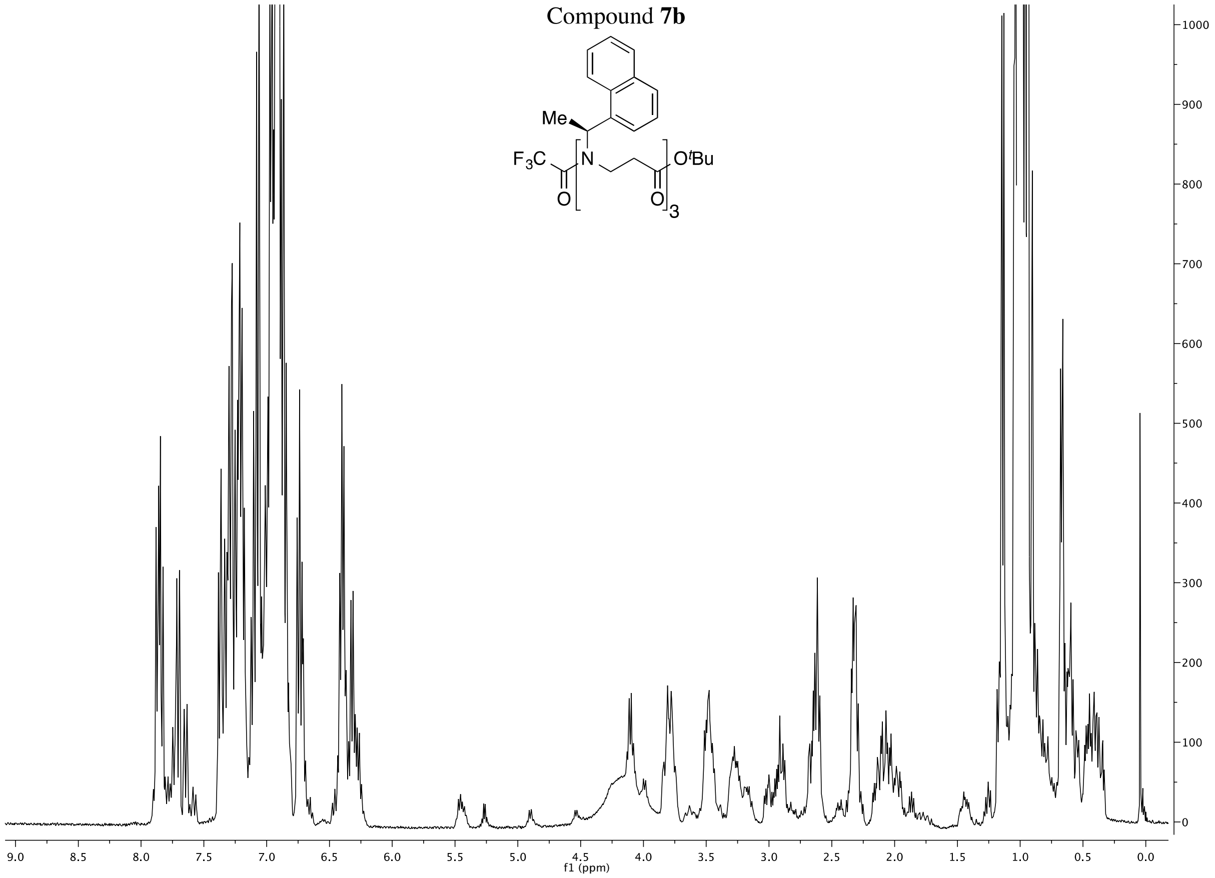

Supplementary Figure 37 | ¹H NMR spectrum of compound 7b. The spectrum was recorded in benzene-d_6 at room temperature.

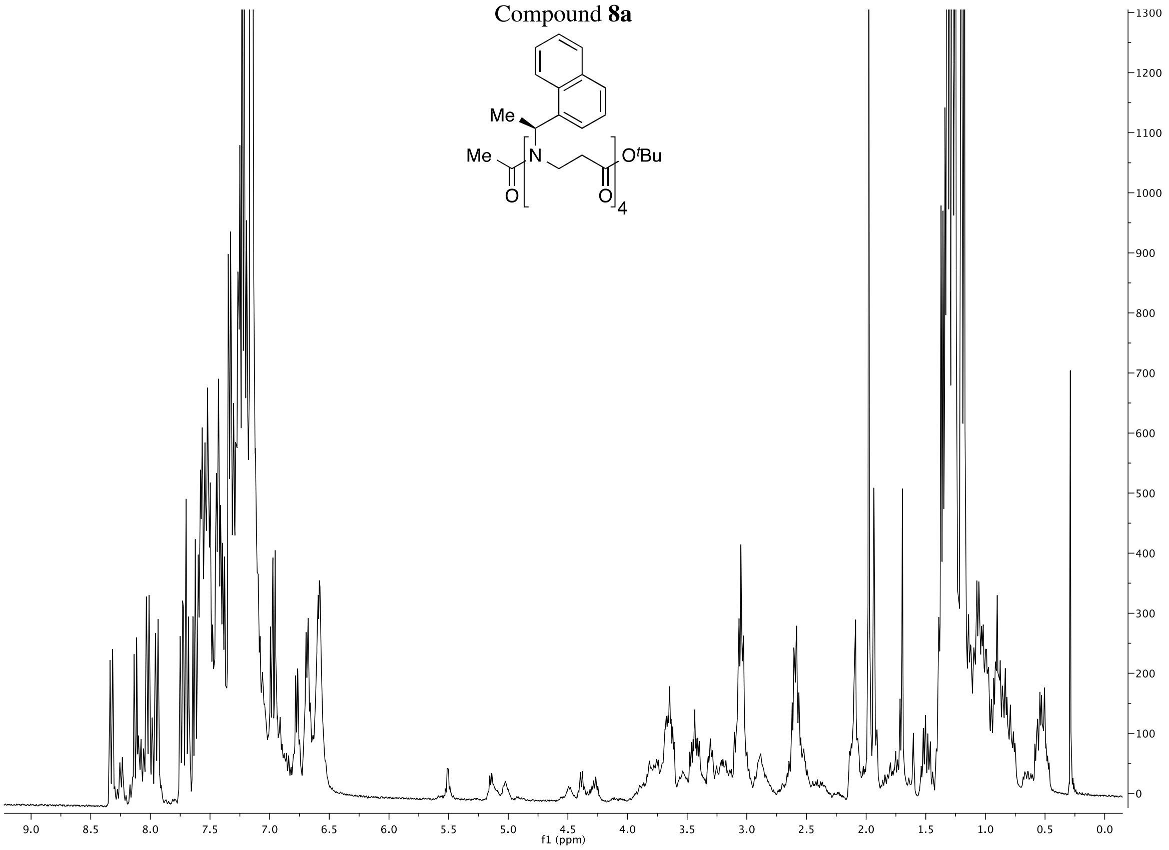

Supplementary Figure 38 | ^{1}H NMR spectrum of compound 8a. The spectrum was recorded in benzene-d_6 at room temperature.

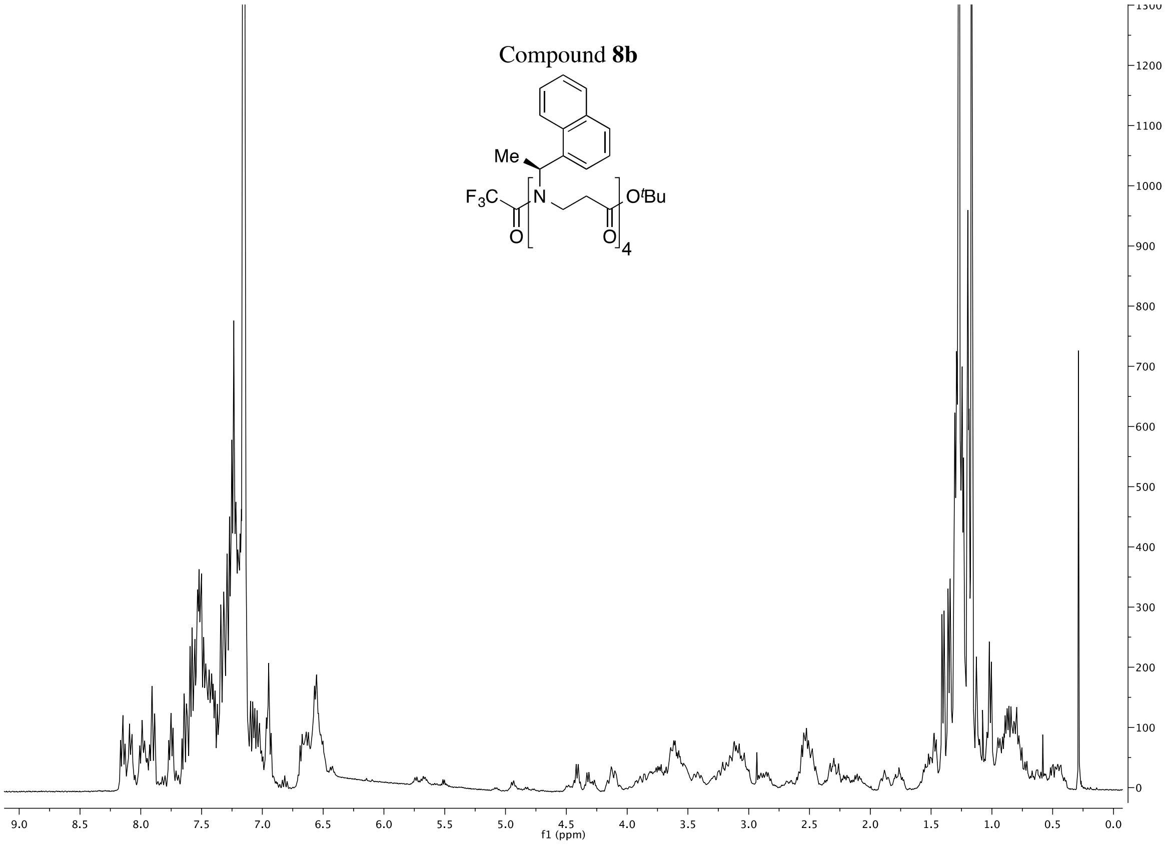

Supplementary Figure 39 | ^{1}H NMR spectrum of compound 8b. The spectrum was recorded in benzene-d_6 at room temperature.

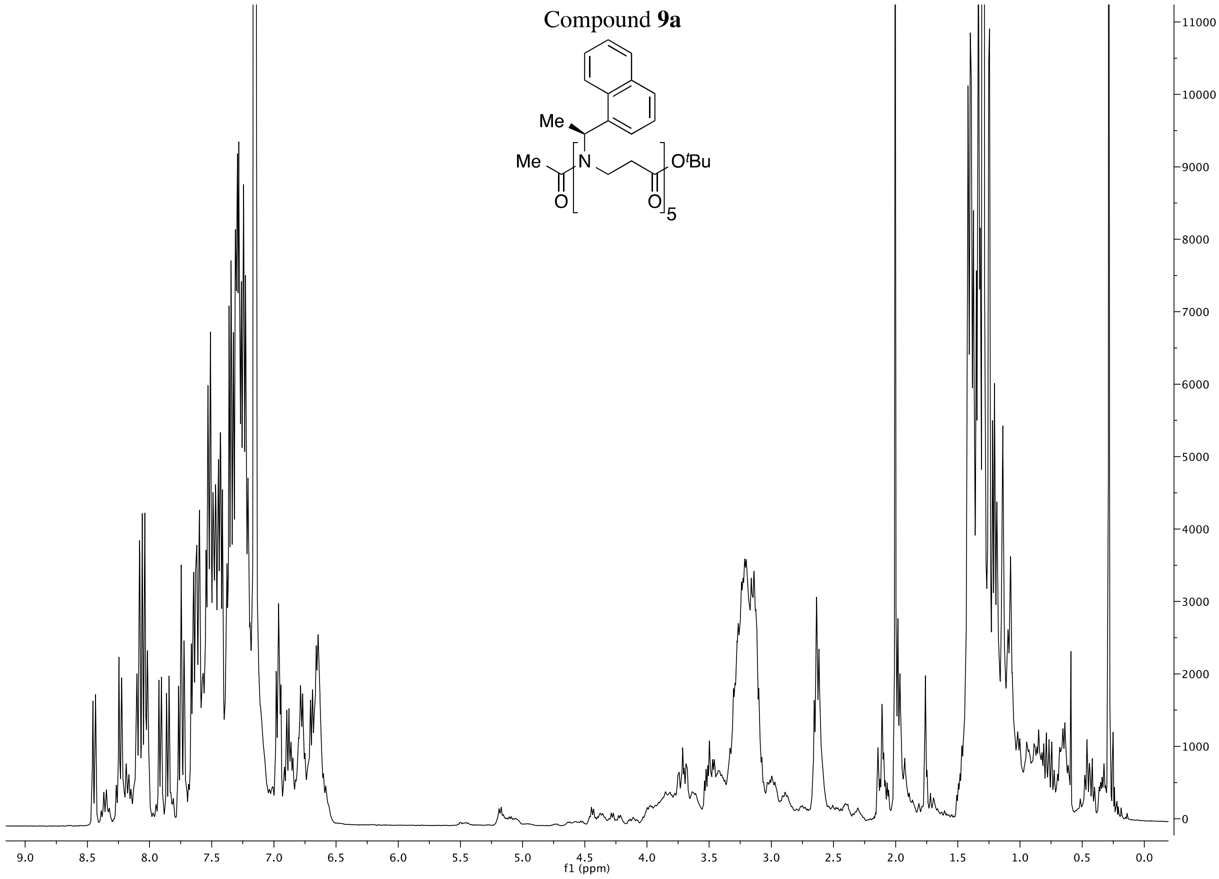

Supplementary Figure 40 | ^{1}H NMR spectrum of compound 9a. The spectrum was recorded in benzene-d_6 at room temperature.

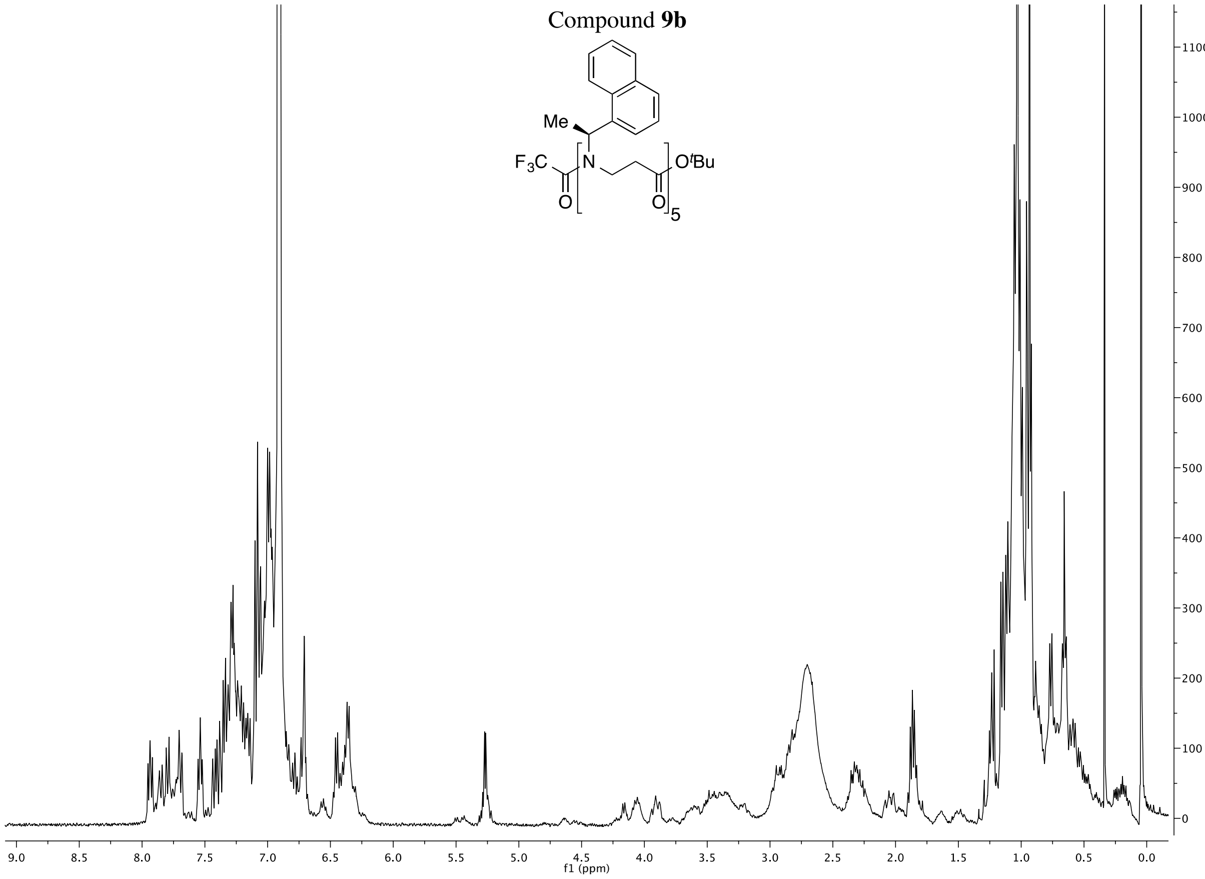

Supplementary Figure 41 | ^{1}H NMR spectrum of compound 9b. The spectrum was recorded in benzene-d_6 at room temperature.

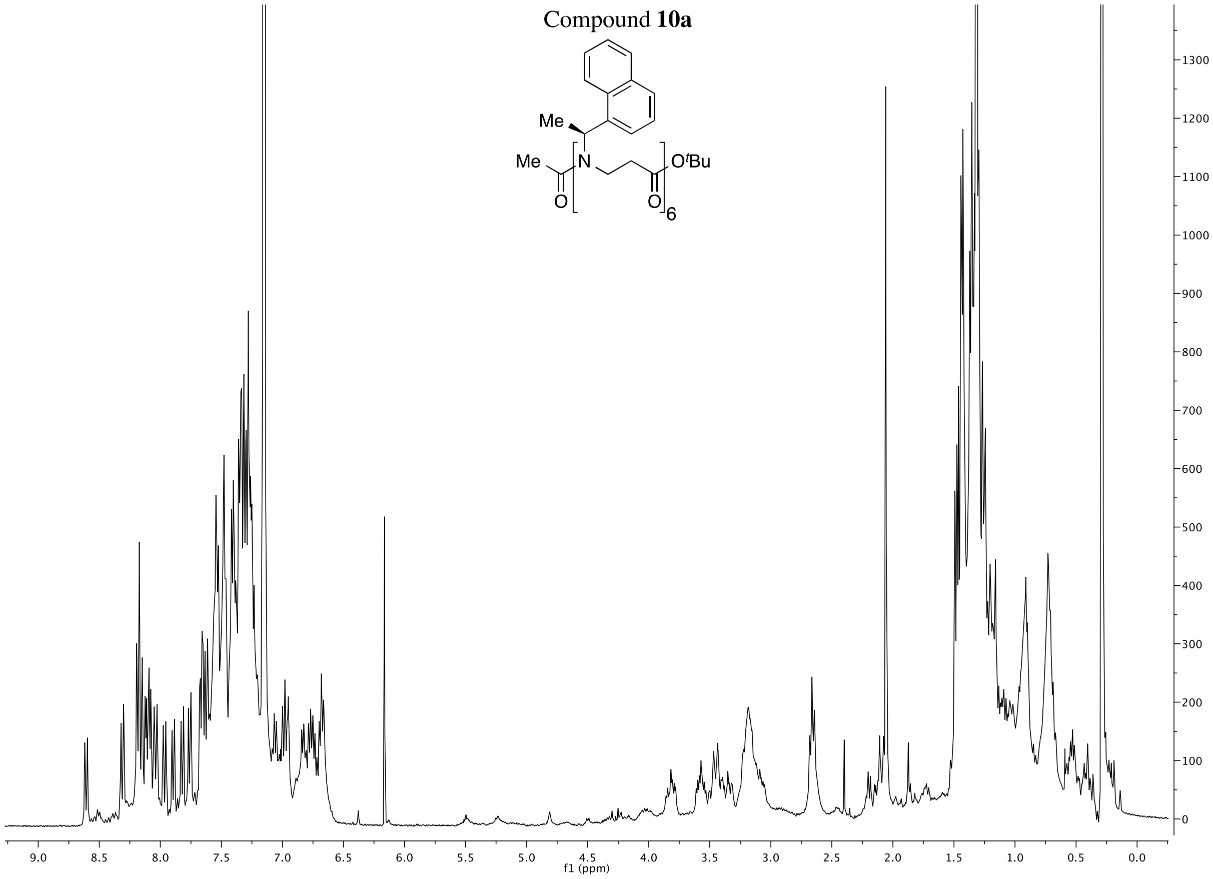

Supplementary Figure 42 | ^{1}H NMR spectrum of compound 10a. The spectrum was recorded in benzene-d_6 at room temperature.

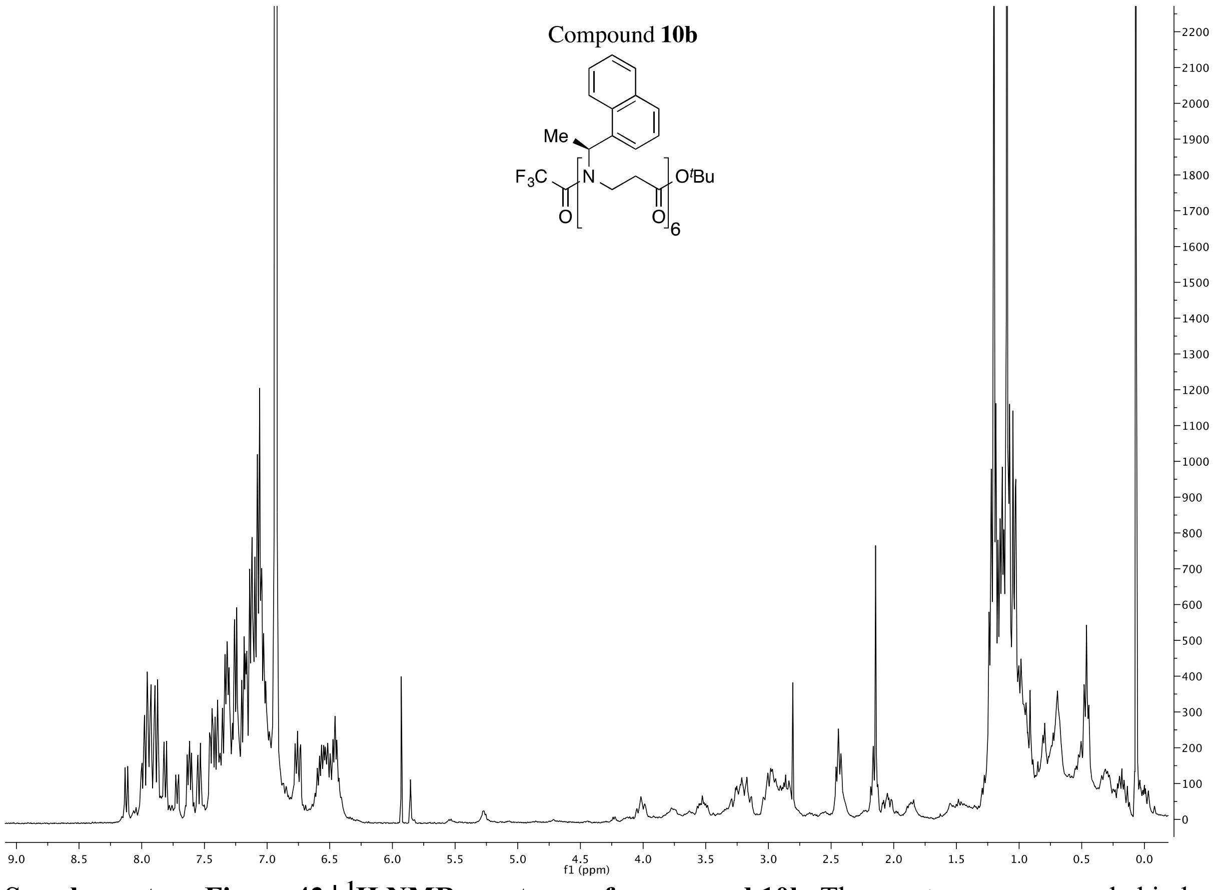

Supplementary Figure 43 | ^{1}H NMR spectrum of compound 10b. The spectrum was recorded in benzene-d_6 at room temperature.

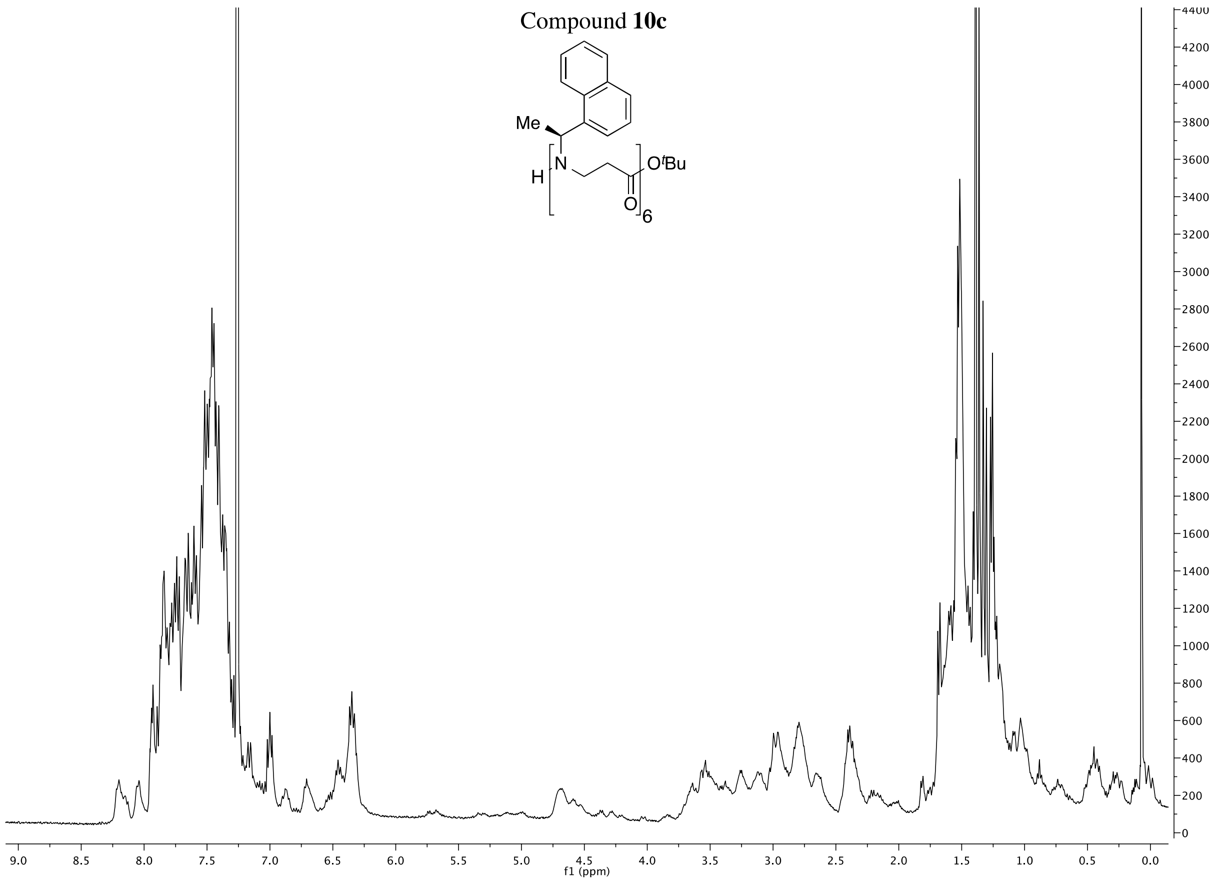

Supplementary Figure 44 | ^{1}H NMR spectrum of compound 10c. The spectrum was recorded in chloroform-d_3 at room temperature.

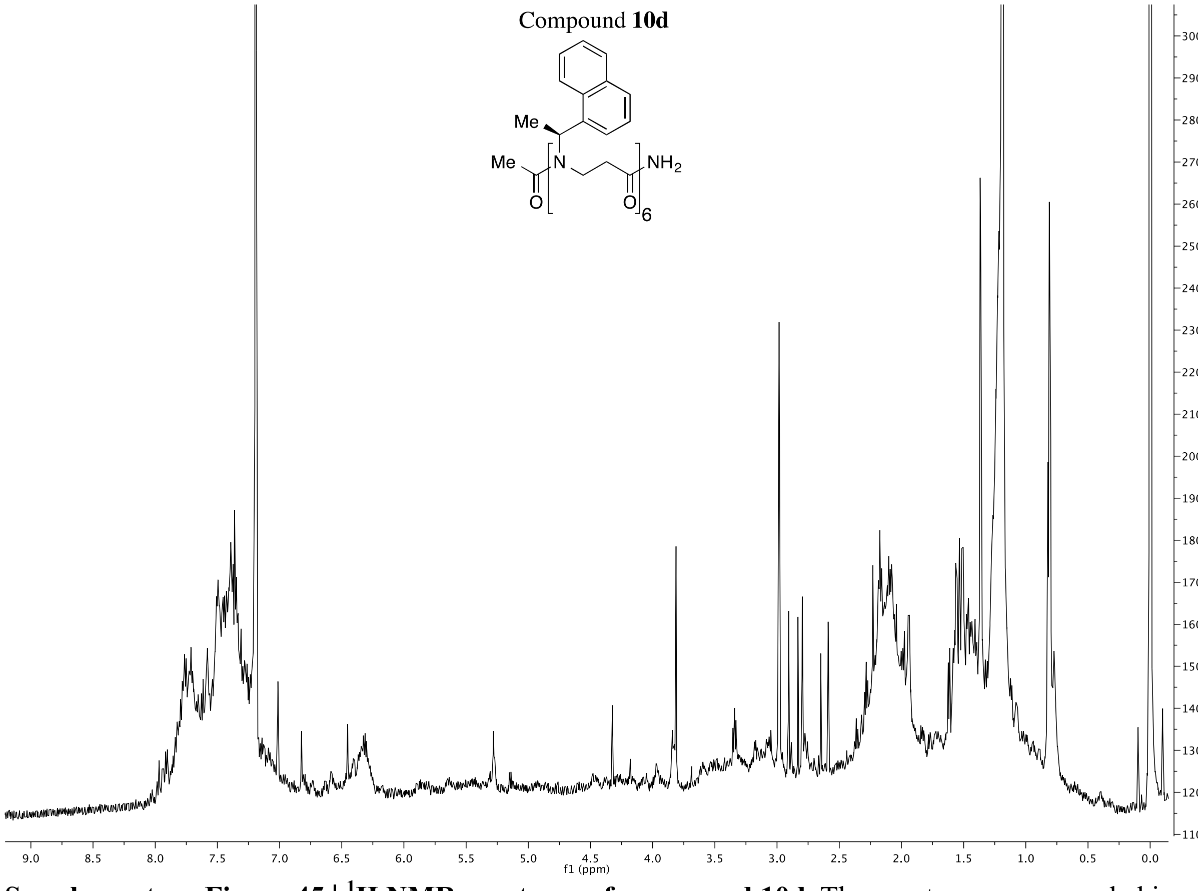

Supplementary Figure 45 | ^{1}H NMR spectrum of compound 10d. The spectrum was recorded in chloroform-d_3 at room temperature.

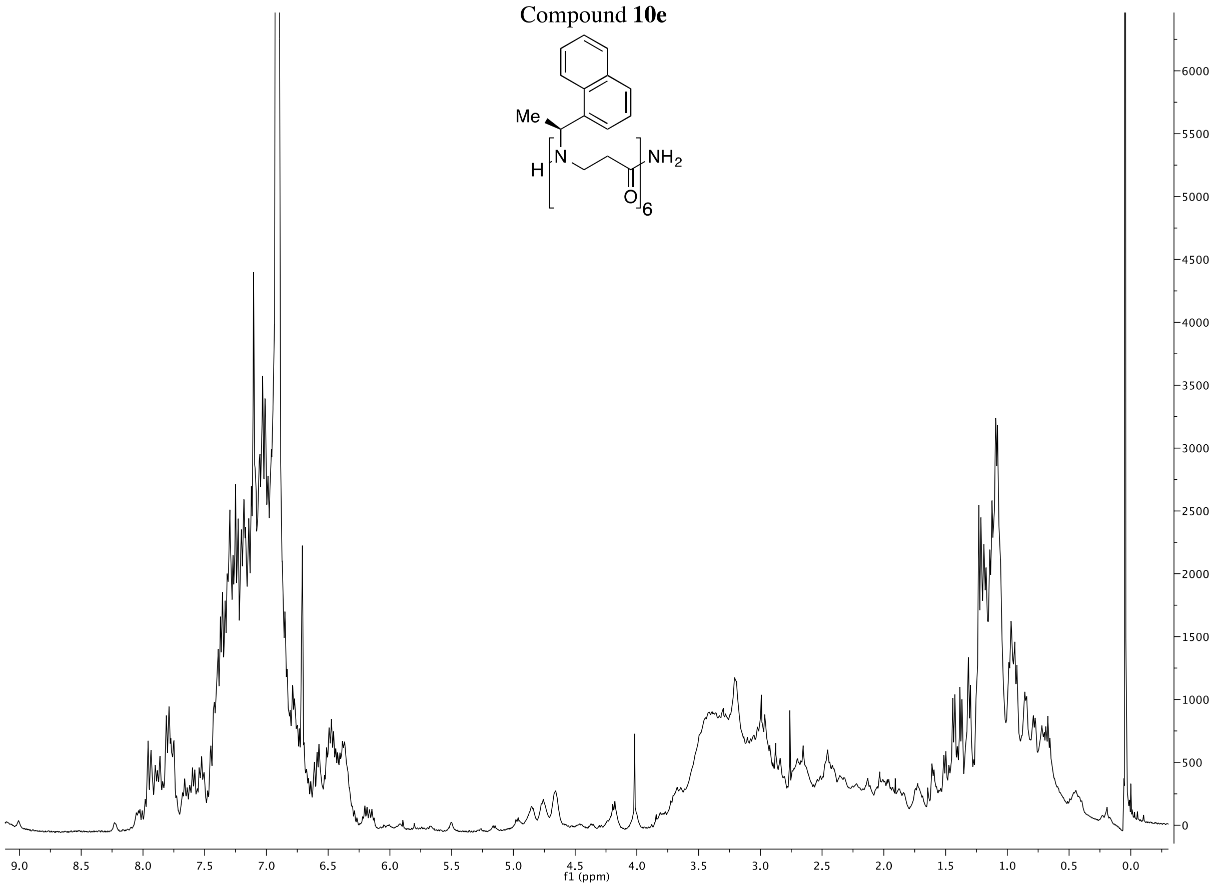

Supplementary Figure 46 | ¹H NMR spectrum of compound 10e. The spectrum was recorded in benzene-d_6 at room temperature.

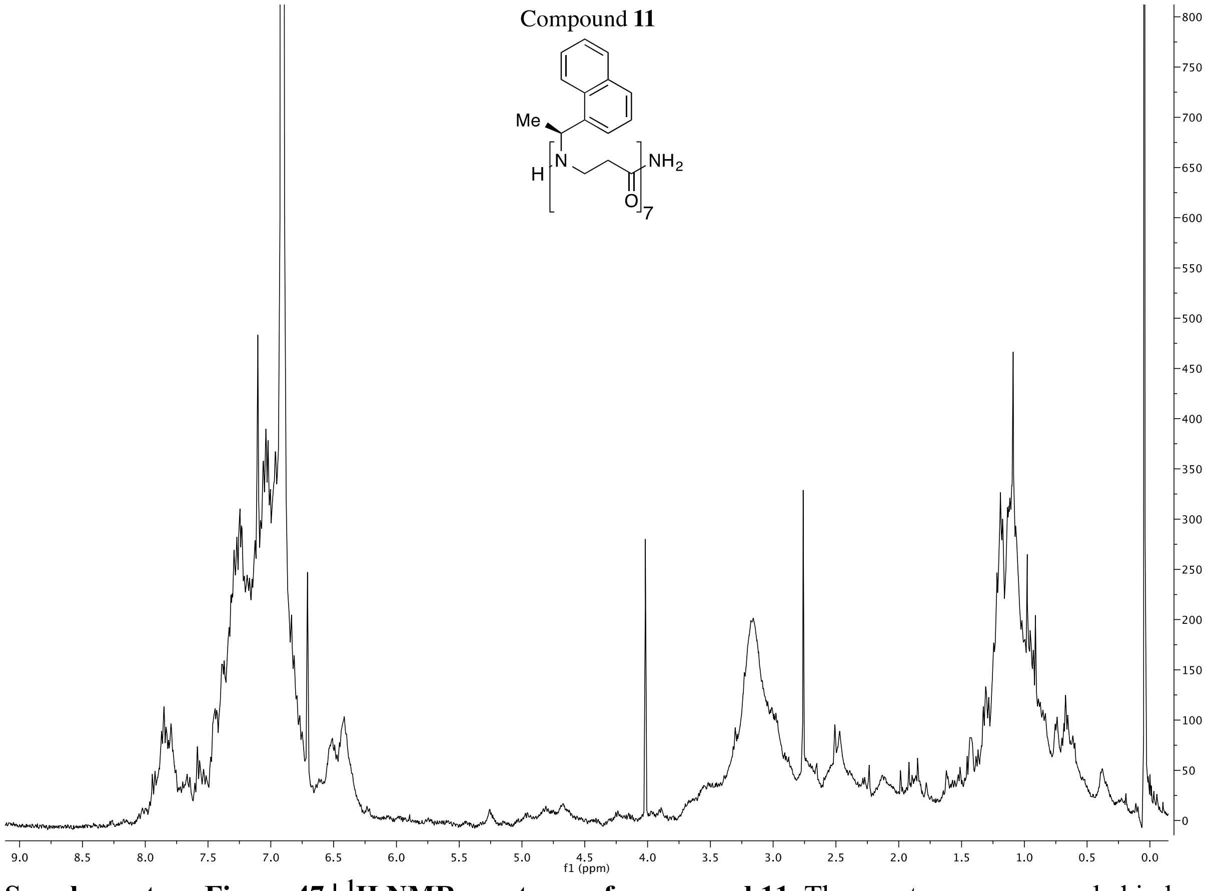

Supplementary Figure 47 | ¹H NMR spectrum of compound 11. The spectrum was recorded in benzene-d_6 at room temperature.

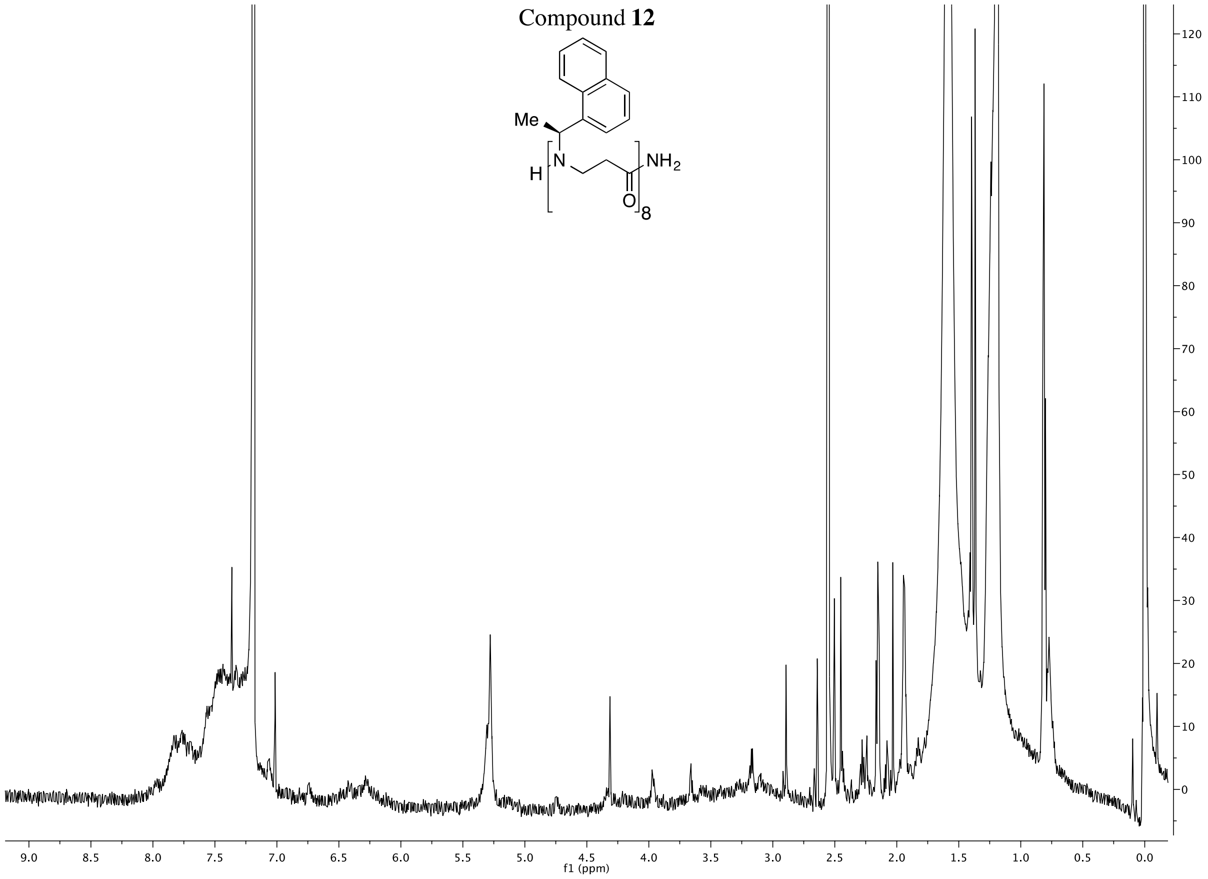

Supplementary Figure 48 | ^{1}H NMR spectrum of compound 12. The spectrum was recorded in chloroform-d_3 at room temperature.

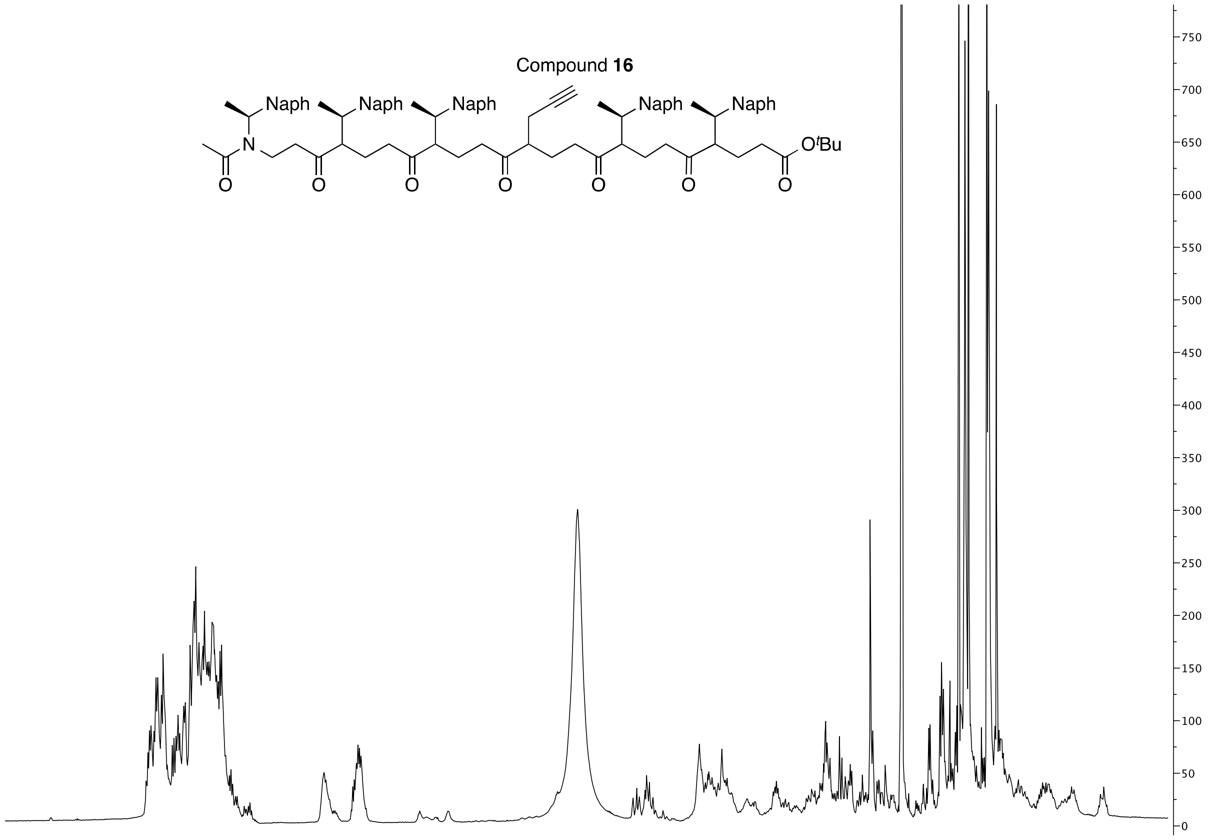

Supplementary Figure 49 | ^{1}H NMR spectrum of compound 16. The spectrum was recorded in acetonitrile-d_3 at room temperature.

Supplementary Tables

Supplementary Table 1 | Torsion angles for additional residues

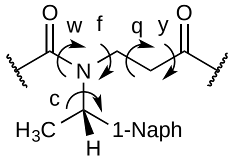

Compound **10a**						
2	ϕ	θ	ψ	ω	χ1	χ2
	96.2	172.5	–175.3	–13.7	53.6	–80.5
Compound **10c**						
Residue	φ	ϕ	ψ	ω	χ1	χ2
3	96.9	166.4	–179.9	–19.4	66.7	–61.5
4	94.3	168	–178.8	–9.5	54.4	–74.2
5	97.4	166	–173.9	–13.4	56.3	–73.8
From DFT calculations previously published (Hofmann et al.)[2]						
	95.8	–179.6	–178.2	8.6	n.a.	n.a.

Supplementary Table 2 | Examples of naphthyl-backbone interactions[a]

Compound **10a**	Distance (Å)	Compound **10c**	Distance (Å)
C1–C6	3.31(2)	C22–C62	3.35(2)
C3–C15	□□□□□□□	C14–C152	3.40(2)
O1–C15	3.54(1)	C152–O14	3.53(2)

[a]Shown in green in Supplementary Fig. 9.

Supplementary Table 3 | Characterization data for compounds 1a–10c

Comp.	Formula	Yield[a] [%]	MS: m/z for $[M+H]^+$ [calcd./obs.]	HRMS: m/z for $[M+H]^+$ [calcd./obs. (ΔM)]	HPLC [t_R/min]	HPLC [% Purity][b]
**1a**	$C_{28}H_{38}N_2O_4$	21 (21)	467.3/467.3	467.2904/467.2920 (3.4)	12.50	96
**1b**	$C_{28}H_{35}N_2O_4$	30 (30)	521.3/521.2	521.2622/521.2630 (1.6)	14.11	95
**2a**	$C_{39}H_{51}N_3O_5$	35 (36)	642.4/642.5	642.3901/642.3901 (0.0)	13.32	95
**2b**	$C_{39}H_{48}F_3N_3O_5$	25 (16)	696.4/696.4	696.3619/696.3625 (0.9)	15.10	98
**3a**	$C_{50}H_{64}N_4O_6$	34 (16)	817.5/817.5	839.4718/839.4737[c] (2.3)	14.24	93
**3b**	$C_{50}H_{61}F_3N_4O_6$	45 (21)	871.5/871.5	871.4616/871.4658 (4.8)	16.06	95
**4a**	$C_{61}H_{77}N_5O_7$	22 (7)	992.6/992.8	992.5896/992.5935 (4.0)	15.00	97
**4b**	$C_{61}H_{74}F_3N_5O_7$	34 (11)	1046.6/1046.7	1046.5613/1046.5646 (3.1)	16.83	95
**5a**	$C_{72}H_{90}N_6O_8$	44 (10)	1167.9/1167.8	1189.6712/1189.6737[c] (2.1)	16.23	98
**5b**	$C_{72}H_{87}F_3N_6O_8$	43 (9)	1221.7/1221.7	1243.6430/1243.6429[c] (0.1)	18.42	97
**6a**	$C_{36}H_{42}N_2O_4$	38 (33)	567.3/567.4	567.3218/567.3242 (4.3)	14.05	96
**6b**	$C_{36}H_{39}F_3N_2O_4$	36 (32)	621.3/621.2	621.2935/621.2959 (3.8)	16.14	99
**7a**	$C_{51}H_{57}N_3O_5$	39 (12)	792.4/792.4	792.4371/792.4396 (3.1)	18.40	95
**7b**	$C_{51}H_{54}F_3N_3O_5$	16 (11)	846.4/846.3	846.4089/846.4114 (3.3)	19.41	98
**8a**	$C_{66}H_{72}N_4O_6$	30 (18)	1017.6/1017.5	1017.5526/1017.5566 (3.9)	20.34	99
**8b**	$C_{66}H_{69}F_3N_4O_6$	32 (19)	1071.5/1071.5	1071.5242/1071.5287 (4.2)	23.17	97
**9a**	$C_{81}H_{87}N_5O_7$	34 (10)	1242.7/1242.6	1264.6498/1264.6537[c] (3.2)	24.31	96
**9b**	$C_{81}H_{84}F_3N_5O_7$	6 (2)	1296.6/1296.4	648.8234/648.8230[d] (0.6)	22.14	97
**10a**	$C_{96}H_{102}N_6O_8$	53 (14)	1468.9/1468.7	734.8969/734.8985[d] (2.1)	28.66	96
**10b**	$C_{96}H_{99}F_3N_6O_8$	54 (15)	1522.9/1522.4	761.8828/761.8832[d] (0.5)	30.06	95
**10c**	$C_{94}H_{100}N_6O_7$	60	1426.7/1426.6	1432.7191/1432.7222 (2.2)	14.16	92

[a]Isolated yields for addition of one sub-unit and subsequent acylation (3 steps), overall yields are given in parentheses. [b]The HPLC purities are reported at 230 nm. [c]The values correspond to $[M+Na]^+$. [d]The values correspond to $[M+2H]^{2+}$

Supplementary Table 4 | Characterization data for compounds 10d–12

Comp	Formula	Yield	MS: m/z for $[M+H]^+$ (calcd./obs.)	HRMS: m/z for $[M+Na]^+$ [calcd./obs. (ΔM)]	HPLC [t_R/min]	HPLC [%Purity][a]
**10d**	$C_{92}H_{95}N_7O_7$	2.0	1410.7/1410.7	1425.7726/1425.7697 (3.4)	16.59	>95
**10e**	$C_{92}H_{95}N_7O_7$	8.2	1369.8 / 1369.8	1390.7080/1390.7080 (0.0)	11.51	>95
**11**	$C_{107}H_{110}N_8O_8$	6.4	1595.1 / 1595.8	1616.8267/1616.8265 (0.1)	12.26	>95
**12**	$C_{122}H_{125}N_9O_9$	3.5	1820.4 / 1820.8	1841.9420/1841.9424 (0.2)	13.36	>95

[a]The HPLC purities are reported at 230 nm . [b]The values correspond to $[M+2H]^{2+}$

Supplementary References

1. Macrae, C. F., *et al. J. Appl. Crystallogr.* **41,** 466–470 (2008).

2. Baldauf, C., Guenther, R., & Hofmann H.-J., *Phys. Biol.* **3,** S1–S9 (2006).
